# Electrochemical Reduction of Carbon Dioxide to 1‐Butanol on Oxide‐Derived Copper

**DOI:** 10.1002/anie.202008289

**Published:** 2020-09-09

**Authors:** Louisa Rui Lin Ting, Rodrigo García‐Muelas, Antonio J. Martín, Florentine L. P. Veenstra, Stuart Tze‐Jin Chen, Yujie Peng, Edwin Yu Xuan Per, Sergio Pablo‐García, Núria López, Javier Pérez‐Ramírez, Boon Siang Yeo

**Affiliations:** ^1^ Department of Chemistry National University of Singapore 3 Science Drive 3 Singapore 117543 Singapore; ^2^ Solar Energy Research Institute of Singapore National University of Singapore 7 Engineering Drive 1 Singapore 117574 Singapore; ^3^ Institute of Chemical Research of Catalonia The Barcelona Institute of Science and Technology Av. Països Catalans 16 43007 Tarragona Spain; ^4^ Institute for Chemical and Bioengineering Department of Chemistry and Applied Biosciences ETH Zürich Vladimir-Prelog-Weg 1 8093 Zürich Switzerland; ^5^ Department of Chemical and Biomolecular Engineering National University of Singapore 4 Engineering Drive 4 Singapore 117585 Singapore

**Keywords:** 1-butanol, carbon dioxide reduction, density functional theory, electrochemistry, reaction mechanisms

## Abstract

The electroreduction of carbon dioxide using renewable electricity is an appealing strategy for the sustainable synthesis of chemicals and fuels. Extensive research has focused on the production of ethylene, ethanol and *n*‐propanol, but more complex C_4_ molecules have been scarcely reported. Herein, we report the first direct electroreduction of CO_2_ to 1‐butanol in alkaline electrolyte on Cu gas diffusion electrodes (Faradaic efficiency=0.056 %, *j*_1‐Butanol_=−0.080 mA cm^−2^ at −0.48 V vs. RHE) and elucidate its formation mechanism. Electrolysis of possible molecular intermediates, coupled with density functional theory, led us to propose that CO_2_ first electroreduces to acetaldehyde‐a key C_2_ intermediate to 1‐butanol. Acetaldehyde then undergoes a base‐catalyzed aldol condensation to give crotonaldehyde via electrochemical promotion by the catalyst surface. Crotonaldehyde is subsequently electroreduced to butanal, and then to 1‐butanol. In a broad context, our results point to the relevance of coupling chemical and electrochemical processes for the synthesis of higher molecular weight products from CO_2_.

## Graphical abstract

Carbon dioxide was electroreduced to 1‐butanol on oxide‐derived copper. The reaction mechanism was determined to proceed through a combination of electrochemical and chemical steps, some of which require contrasting conditions. This example highlights limitations in one‐pot synthesis and provides a case for utilizing independently optimized sequential reactors in a tandem system to build complex molecules from low molecular‐weight feedstocks.

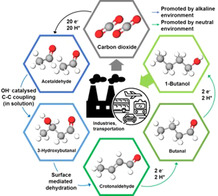

## Introduction

The electrochemical carbon dioxide reduction reaction (CO_2_RR) to fuels and chemicals, when powered by renewable electricity, is a potentially sustainable way to alleviate our pressing global energy demands and to avert climate change.^[1]^ Copper‐based materials are the only family of catalysts that can reduce CO_2_ to multi‐carbon molecules with significant Faradaic efficiencies (*FE*) and current densities (*j*).[^2^, ^3^] Among the multi‐carbon products, C_2_ molecules such as ethylene and ethanol can be facilely formed (*FE*=30–50 %).[^2^, ^4^] The main C_3_ product reported is *n*‐propanol (*FE*=10–13 %),[^5^, ^6^] alongside small quantities of propionaldehyde, allyl alcohol, acetone, propylene and propane (total FE<3 %).[^4^, ^5^] Reports on the formation of C_4_ molecules, many of which have much higher commercial value, are scarce and have been limited to hydrocarbons showing *FE*<1 %.^[7]^ The drastic decrease in the selectivity of a product as the number of carbon atoms in it increases suggests that the coupling mechanism to form a multi‐carbon product follows a “polymerization” scheme of *CO that obeys the Flory‐Schulz distribution.

Interestingly, the direct production of 1‐butanol (CH_3_CH_2_CH_2_CH_2_OH) from electrochemical CO_2_ reduction has not been reported. This oxygenate, which has a high volumetric energy density of 29.2 MJ L^−1^ and is less hygroscopic and corrosive than ethanol, has been suggested for direct use as a fuel or in diesel‐blends.^[8]^ Schmid and co‐workers have utilized bacteria to convert CO (generated from CO_2_ electrolysis) to 1‐butanol.^[9]^ More recently, a mechanistic study of CO_2_ reduction to *n*‐propanol revealed that minor and yet‐to‐be‐quantified amounts of 1‐butanol can be co‐produced from the electrochemical reduction of acetaldehyde and CO in 0.1 m KOH on oxide‐derived Cu electrodes.^[10]^ Still, no strategy to successfully electrosynthesize 1‐butanol or any C_4_ oxygenates from CO_2_ has been conceived. To tackle this challenge, it is crucial to understand and map out the mechanism and kinetics for its formation.

**Figure 1:**
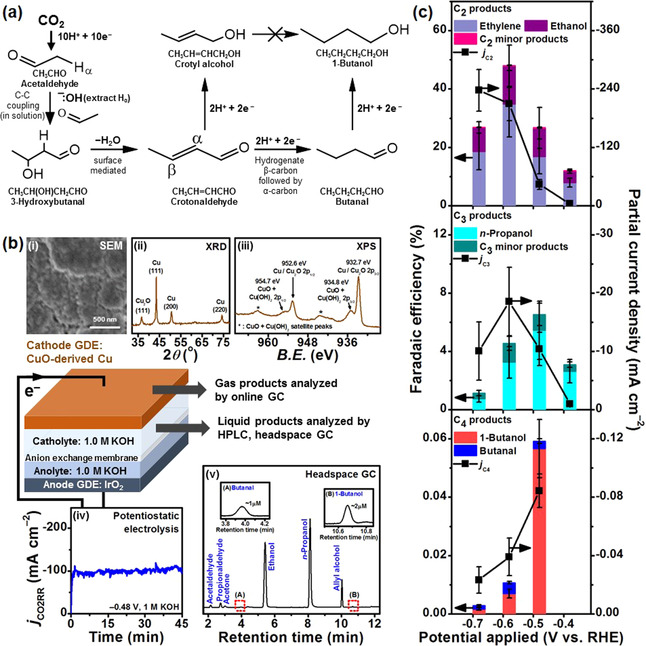
a) Simplified reaction scheme for CO_2_ reduction to 1‐butanol. Further details are provided in Section S3. b) Diagram of CO_2_ flow cell electrolysis set up: characterization of CuO‐derived Cu cathodes by (i) SEM, (ii) XRD and (iii) XPS (*B.E*. refers to binding energy). We note that the detection of Cu_2_O in the XRD and CuO + Cu(OH)_2_ signals in the XPS data are likely due to surface oxidation of the electrode from its exposure to air (see Section S2.1 for a more detailed discussion); (iv) large CO_2_ reduction current densities (in the order of −100 mA cm^−2^) which gave a sufficient rate of product formation to allow detection of minor products; (v) sensitive analytical techniques like headspace GC can quantify minor products down to the μm‐scale. c) Faradaic efficiencies (*FE*, colored bars) and partial current densities (*j*, ▪) of C_2_, C_3_ and C_4_ products from electrolysis of CO_2_ on CuO‐derived Cu GDE in 1.0 m KOH. The major C_2_, C_3_ and C_4_ products are ethylene, *n*‐propanol and 1‐butanol, respectively. Other detected products are shown in Table S1.

Herein, we report and quantify for the first time the formation of C_4_ oxygenates from alkaline electrolysis of CO_2_ using CuO‐derived Cu gas diffusion electrodes (GDE) in a flow cell. The predominant C_4_ product was 1‐butanol (*FE*=0.056 %, *j*_1‐Butanol_=−0.080 mA cm^−2^) at −0.48 V vs. RHE (Reversible Hydrogen Electrode; from here on, all potentials are referenced to the RHE and all currents are normalized to the exposed geometric surface area of the electrodes, unless otherwise stated). We then elucidate the reaction mechanism by combining analyses of reaction products from electrolyses of possible intermediates and density functional theory (DFT) investigations. The formation of the critical C_4_ intermediate, crotonaldehyde (CH_3_CH=CHCHO), was traced to the aldol condensation (C−C bond formation) of two acetaldehyde (CH_3_CHO) molecules generated from CO_2_ electroreduction (Figure 1 a). The aldol reaction is promoted by both OH^−^ ions in the electrolyte and the electrocatalyst surface. Crotonaldehyde then undergoes a two‐step electroreduction to 1‐butanol. We also unveil the critical role of pH at different stages of the reaction mechanism, pointing towards new strategies for increasing the performance of electro‐assisted conversion of CO_2_ into 1‐butanol.

## Results and Discussion

We electroreduced CO_2_ at various potentials using CuO‐derived Cu GDE cathodes in a flow cell (Figure 1 b, Sections S1,S2). The catalyst was electrodeposited onto the GDE using a previously‐published procedure.^[11]^ Aqueous 1.0 m KOH was used as the electrolyte. The high CO_2_RR current densities from the flow cell electrolysis (Figure 1 b(iv)), which circumvents mass transport limitations, combined with the use of highly sensitive headspace gas chromatography (Figure 1 b(v)) improves the detection and quantification of liquid products with low *FEs* and current densities. This allows us to detect CO_2_ reduction products that have, to‐date, never been observed.

The total *FEs* of carbonaceous products were 68‐69 % at −0.48 and −0.58 V (Figure 1 c, Table S1). The major multi‐carbon products are C_2_ molecules, namely ethylene and ethanol, which are typically formed on oxide‐derived copper catalysts.[^2^, ^12^] The highest *FE* ${}_{C{}_{2}}$ of 48 % was observed at −0.58 V, with a corresponding *j* ${}_{C{}_{2}}$ of −210 mA cm^−2^. Minor C_2_ products (*FE*≤0.1 %) such as acetaldehyde and ethane were also detected. In the case of acetaldehyde, the low *FE* is a result of the chemical and/or electrochemical transformations it readily undergoes, as we will discuss below. C_3_ species, mainly *n*‐propanol, were also detected, with a maximum *FE* ${}_{C{}_{3}}$ of 6.5 % and *j* ${}_{C{}_{3}}$ of −18.5 mA cm^−2^ obtained at −0.48 and −0.58 V, respectively. Overall, the catalytic activities toward C_2_ and C_3_ molecules from CO_2_ reduction are comparable or higher than values previously reported for Cu catalysts loaded onto carbon GDEs (Table S3). Interestingly, we detected C_4_ oxygenates such as 1‐butanol and butanal (maximum total *FE* ${}_{C{}_{4}}$ =0.060 % at the onset potential of −0.48 V), in contrast to previous studies which only identified hydrocarbons for the C_4_ fraction.[^7^, ^13^] The dominant product was 1‐butanol, which is in line with the fact that aldehydes can be easily electroreduced to their corresponding alcohols, as demonstrated in the cases of formaldehyde and acetaldehyde.^[14]^ No carbon‐containing products were found in control experiments performed without applied potentials.

As 1‐butanol is the sole C_4_ alcohol product, it could not come from C−C coupling of four individual C_1_ adsorbates such as *CO in a Flory‐Schulz distribution, since 2‐butanol was not detected. This led us to postulate that the formation of 1‐butanol could occur through a combination of electrochemical and chemical steps. Specifically, the aldol condensation of two C_2_ intermediates, such as acetaldehyde, gives rise to the C_4_ backbone of crotonaldehyde, which is further reduced to C_4_ terminal oxygenates like butanal and 1‐butanol. While mechanisms for the formation of major C_2_ and C_3_ products, including acetaldehyde, have been widely discussed in the literature,[^6^, ^10^, ^15^, ^16^, ^17^, ^18^] pathways for producing C_4_ products are rarely mentioned. Herein, we focus on acetaldehyde reactivity as it is much less explored mechanistically. Nonetheless, we also present the two steps preceding acetaldehyde formation, which involve the key ethenyloxy intermediate, CH_2_CHO*, and discuss the lateral pathways for CO_2_ reduction to C_1_‐C_3_ products in Section S3.

**Figure 2:**
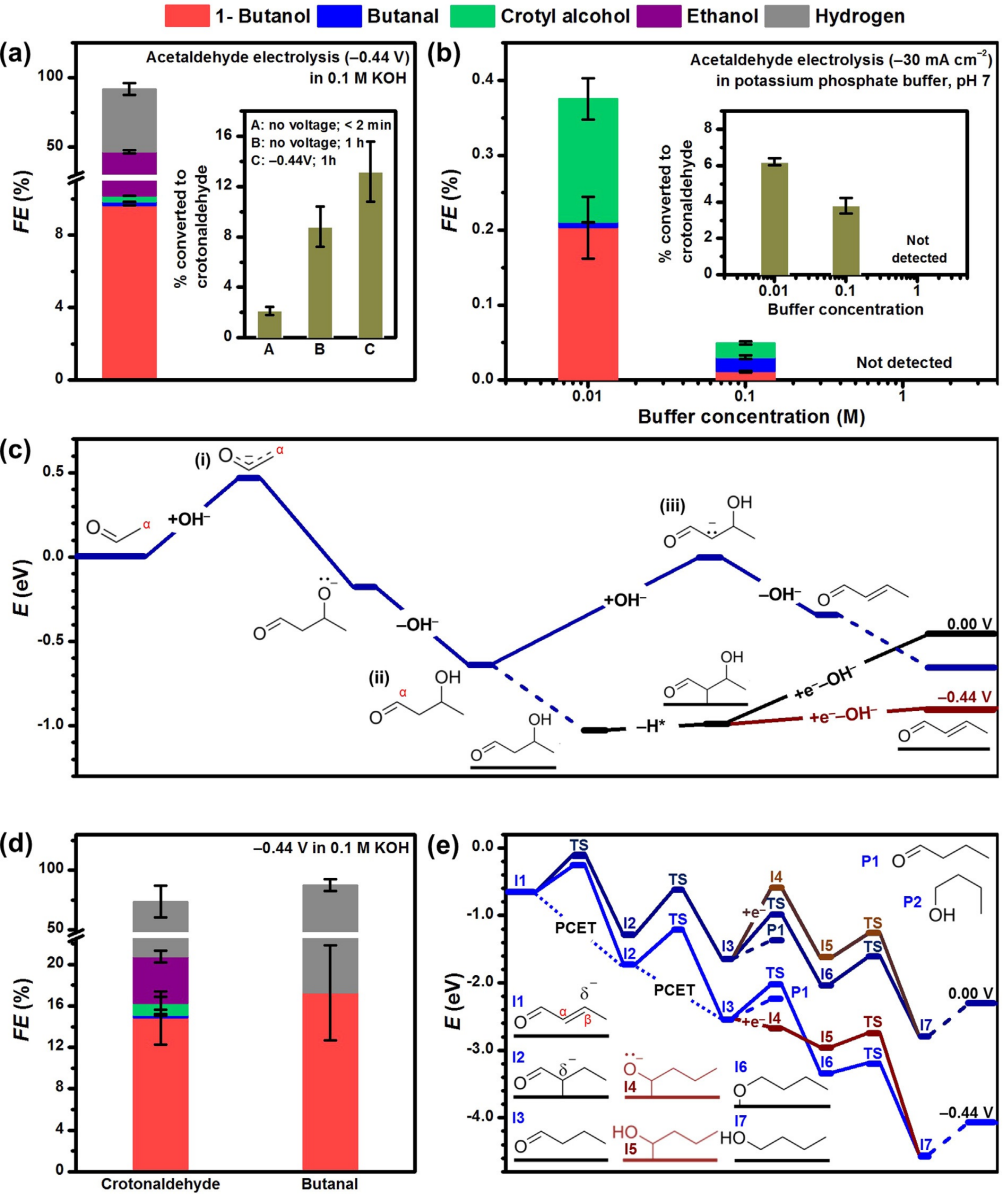
Faradaic efficiencies of products from 1 h electrolysis of 50 mm acetaldehyde on CuO‐derived Cu (a) in alkaline and (b) neutral buffer (62 mol % K_2_HPO_4_ + 38 mol % KH_2_PO_4_) electrolyte. The insets of (a) and (b) show the percentage of acetaldehyde that was converted to crotonaldehyde in the electrolyte in each case. c) Potential energy diagram of acetaldehyde condensation to crotonaldehyde in solution (dark blue) and mediated by the surface (black). The final OH^−^ removal, which can be assisted by one electron donated from the surface, is promoted by reductive potentials (dark red). Water molecules were omitted for clarity. d) Faradaic efficiencies of products from alkaline electrolyses of 50 mm crotonaldehyde and 50 mm butanal on CuO‐derived Cu. e) Potential energy diagram of crotonaldehyde reduction to 1‐butanol via butanal (blue). Under negative potentials, butanal can be adsorbed via a one‐electron transfer (dark red), which promotes its further reaction instead of desorption. Dashed lines represent adsorptions/desorptions. Dotted lines represent proton‐coupled electron transfers (PCET). Additional (electro)chemical routes are shown in Sections S3 and S6.

To test our hypothesis for 1‐butanol formation via acetaldehyde, we electroreduced 50 mm acetaldehyde in 0.1 m KOH on CuO‐derived Cu electrodes (Section S4). The product distribution at an optimized potential of −0.44 V is summarized in Figure 2 a (see also Table S5 for product distributions at other applied potentials). The product with the highest selectivity was 1‐butanol (*FE*=9.6 %, *j*_1‐Butanol_=−1.06 mA cm^−2^), consistent with expectations from a base‐catalyzed aldol condensation C−C coupling step. The remaining electrolysis products were other C_4_ oxygenates such as butanal (CH_3_CH_2_CH_2_CHO) and crotyl alcohol (CH_3_CH=CHCH_2_OH), as well as ethanol. During the electrolysis, we observed some coloration of the anion‐exchange membrane due to its exposure to the alkaline acetaldehyde‐containing electrolyte, but control experiments excluded its interference with our electrolysis results (Figures S8,9).

Detection of crotonaldehyde (3.3 mm, equivalent to 13.2 % acetaldehyde conversion) after electrolysis suggests that the C_4_ backbone of 1‐butanol could be formed via a base‐catalyzed aldol condensation (Figure 2 a, inset). We utilized the increase in local pH close to the electrode under electroreduction conditions to test this hypothesis and performed fixed‐current electrolyses of 50 mm acetaldehyde in 0.01, 0.1 and 1.0 m potassium phosphate buffer (pH 7, Figure 2 b, Section S4.3). Higher buffer concentrations mitigate the local pH increase during electroreduction, thus lowering the overall local pH. Our results reveal that electrolysis in 0.01 m potassium phosphate buffer gave the highest selectivity toward C_4_ products (*FE*=0.4 %, with 1‐butanol as the main product) and percentage of acetaldehyde converted to crotonaldehyde (1.5 mm, equivalent to 6.2 % acetaldehyde conversion). In contrast, neither C_4_ products nor crotonaldehyde were detected from experiments performed in 1.0 m buffer (Figure 2 b inset). These observations of an alkaline local pH promoting the production of 1‐butanol on copper directly support its formation via the aldol condensation of two acetaldehyde molecules and suggest an enhanced reaction rate close to the catalytic surface under electrolysis conditions.

We further investigated, using DFT, the base‐catalyzed aldol condensation mechanism to form the C_4_ backbone. High‐level methods including solvent and potential effects have been employed to study electrochemical networks up to C_2_ species, including the formation of acetaldehyde from CO_2_.^[16]^ However, the multiple conformations of C_4_ molecules and the complexity of the reaction network with chemical (bulk solvent and interface) and electrochemical steps limits us to the use of DFT coupled to the Computational Hydrogen Electrode (CHE). The formation of the C_4_ backbone comprises two steps: the C−C coupling between two acetaldehyde molecules to form 3‐hydroxybutanal (CH_3_CH(OH)CH_2_CHO), and the subsequent dehydration of the latter to crotonaldehyde (Figure 2 c). In solution, the aldol condensation starts with the stripping of an α‐hydrogen from acetaldehyde by OH^−^ to form an ethenyloxy anion (CH_2_CHO^−^, Figure 2 c(i)). The α‐carbon of CH_2_CHO^−^ then attacks the carbonyl group of a second acetaldehyde molecule, which is subsequently protonated to 3‐hydroxybutanal (Figure 2 c(ii)). Then, 3‐hydroxybutanal loses an α‐hydrogen as a proton, to generate a carbene species (Figure 2 c(iii)), which forms crotonaldehyde by hydroxyl elimination. Consistent with findings from the literature,^[19]^ the latter is the rate‐determining step in solution. We further note that for the case of CO_2_ reduction, adsorbed ethenyloxy species is formed as a precursor of acetaldehyde and ethanol,[^16^, ^20^] and thus can also readily react with an acetaldehyde molecule in solution and be subsequently hydrogenated to form 3‐hydroxybutanal (Section S3).

The amount of acetaldehyde converted to crotonaldehyde during electrolysis (13.2 %) is larger than the 8.8 % conversion (or 2.2 mm crotonaldehyde) when 50 mm acetaldehyde was aged in 0.1 m KOH for 1 h without applied potential (inset in Figure 2 a). This observation suggests that the Cu surface can promote the aldol condensation at cathodic potentials. DFT analysis reveals that this alternative pathway starts with 3‐hydroxybutanal adsorbing exothermically on Cu and losing an α‐hydrogen as an adsorbed H (dark red in Figure 2 c). The hydroxyl group is then eliminated, in parallel with an electron transfer, to give crotonaldehyde. On the Cu surface, this step is promoted by negative applied potentials. Overall, the DFT investigation reveals that the alkaline electrolyte promotes the initial C−C coupling step between two acetaldehyde molecules, while the Cu surface promotes the subsequent dehydration step to crotonaldehyde.

To elucidate the fate of C_4_ species after the aldol condensation step, we electrolyzed 50 mm crotonaldehyde on CuO‐derived Cu in 0.1 m KOH at −0.44 V (Figure 2 d, Section S5). The major carbonaceous product was 1‐butanol (*FE*=14.8 %), while small amounts of butanal (*FE*=0.3 %) and crotyl alcohol (*FE*=1.1 %) were also detected. The Faradaic selectivity of 1‐butanol (*FE*_1‐Butanol_ normalized by the *FE* of all the C_4_ products) from crotonaldehyde electrolysis was 91.4 %, which is similar to the case of acetaldehyde (94.7 %, Table S10). This reinforces the role of crotonaldehyde as the main intermediate in the electrosynthesis of acetaldehyde to C_4_ oxygenates. The presence of ethanol (*FE*=4.5 %) was attributed to the reduction of acetaldehyde present due to the hydroxide‐catalyzed retro‐aldol reaction of crotonaldehyde, which is known to occur at room temperature.^[21]^ This observation highlights the complexity of crotonaldehyde chemistry under aqueous alkaline conditions, and leads us to infer that the low total Faradaic efficiency of 72.2 % is a consequence of undetected products from other side reactions of crotonaldehyde in the alkaline electrolyte (Figure S10).

Butanal and crotyl alcohol are known intermediates in the gas‐phase hydrogenation of crotonaldehyde to 1‐butanol,^[22]^ and their presence during crotonaldehyde electrolysis suggests that they are potential electrochemically‐active intermediates to 1‐butanol. Therefore, we electrolyzed butanal and crotyl alcohol under the same conditions (Figure 2 d, Table S9). 1‐Butanol was the sole product from the electroreduction of butanal (*FE*=17.3 %; the balance product is H_2_). Only hydrogen was detected during the electrolysis of crotyl alcohol, which indicates that the latter is electrochemically inert, in good agreement with theoretical calculations in Figure S11.

Theoretical analysis of crotonaldehyde reduction reveals that butanal is formed by sequential hydrogenation of the β‐ and α‐carbons of crotonaldehyde (Figure 2 e). Once formed, butanal tends to desorb rather than further react. However, at potentials more reductive than −1.02 V vs. SHE (standard hydrogen electrode), butanal receives an electron from the cathode surface to form the CH_3_CH_2_CH_2_C*HO^−^ anion (I4 in Figure 2 e). As this adsorption does not involve proton transfers, it is independent of the electrolyte pH in the SHE scale. CH_3_CH_2_CH_2_C*HO^−^ is subsequently protonated to yield 1‐hydroxybutyl (I5 in Figure 2 e), which is further hydrogenated in a chemical step (*E*_a_=0.39 eV) to produce 1‐butanol. These theoretical findings are corroborated by our results from crotonaldehyde electrolysis performed at pH 7 and pH 13 (Table S9). At −1.20 V vs. SHE, 1‐butanol was the most selective product in both electrolytes, consistent with the pH‐independent adsorption of butanal. However, at −0.90 V vs. SHE, butanal was the most selective product, indicating that this potential was insufficient for its further reduction to 1‐butanol. Alternative chemical routes from butanal to 1‐butanol are shown in Figure S12.

**Figure 3:**
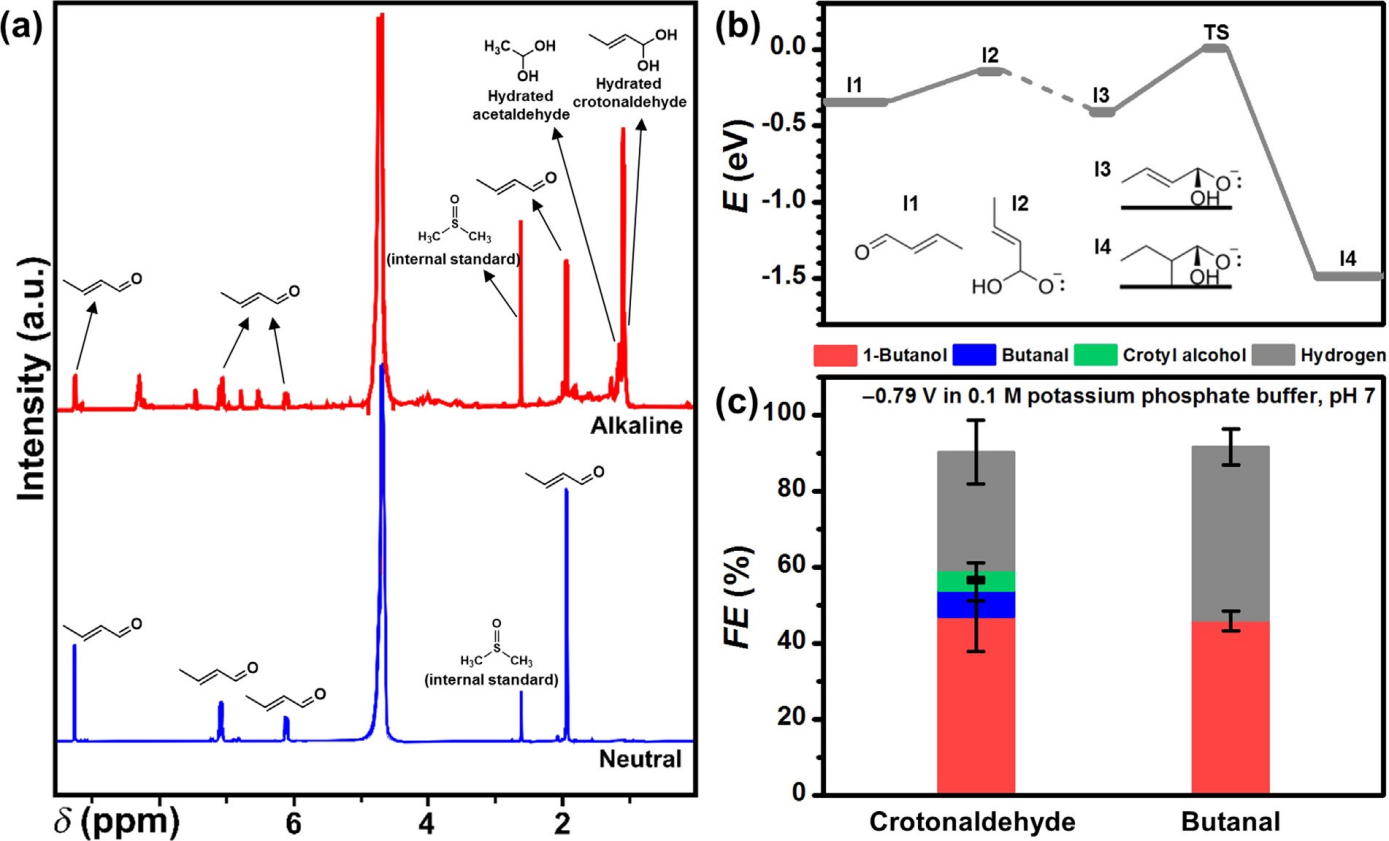
a) ^1^H NMR spectra of 50 mm crotonaldehyde (*δ* refers to the chemical shift) in alkaline environment (0.1 m KOH, red line) and neutral (ultrapure deionized water, blue line). Phenol (7.2 ppm) and dimethylsulfoxide (2.6 ppm) were dissolved in D_2_O (residual H_2_O peak at 4.8 ppm) and added as internal standards. b) Potential energy diagram of crotonaldehyde hydration (I1 to I2) and subsequent hydrogenation. Dashed lines represent adsorption/desorption. c) Faradaic efficiencies of crotonaldehyde and butanal electrolysis products on CuO‐derived Cu in neutral buffer electrolyte.

Aldehydes can be hydrated to geminal diols in aqueous alkaline solution. Signals belonging to hydrated crotonaldehyde (CH_3_CH=CHCH(OH)_2_) were observed in nuclear magnetic resonance (NMR) spectroscopic analyses of 50 mm crotonaldehyde or acetaldehyde dissolved in 0.1 m KOH (Figure 3 a, Figure S13). We therefore considered the possibility of hydrated crotonaldehyde as an intermediate to 1‐butanol. DFT suggests that hydrated crotonaldehyde cannot be further electrochemically reduced due to a larger activation barrier (+0.41 eV) compared to its desorption energy (+0.26 eV), as shown in Figure 3 b. This result was corroborated by the *FE*_1‐Butanol_ of 46‐47 % observed from the crotonaldehyde and butanal electrolyses in 0.1 m potassium phosphate buffer (pH 7) at −0.79 V (Figure 3 c, Table S9), which was almost three times more than the values from electrolyses in 0.1 m KOH. Figure 3 a shows that in a neutral medium, only peaks corresponding to crotonaldehyde were observed. The neutral buffer electrolyte therefore likely suppressed the hydration process and increased the availability of unhydrated crotonaldehyde for reduction.

**Figure 4:**
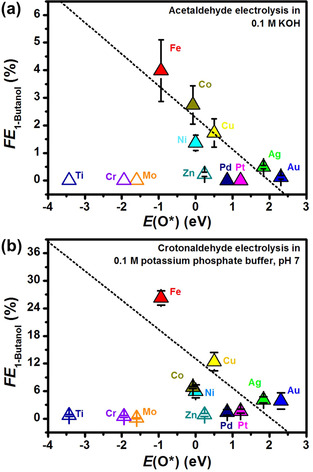
Faradaic efficiencies of 1‐butanol from −10 mA cm^−2^ constant‐current electrolysis of a) acetaldehyde and b) crotonaldehyde on selected metals as a function of the DFT‐computed adsorbed oxygen stability on these metals with respect to water and hydrogen. Metals that are typically oxides at 0 V vs. RHE at the pH of the supporting electrolyte are shown as hollow symbols. 1‐Butanol was not detected from acetaldehyde electrolysis on Ti, Cr, Mo, Pd, and Pt.

To find a better electrocatalyst to enhance the *FE* of 1‐butanol from acetaldehyde and crotonaldehyde reduction, we proceeded to identify an activity descriptor. To this end, we electrolyzed acetaldehyde in 0.1 m KOH and crotonaldehyde in 0.1 m potassium phosphate buffer on different metal discs (Section S8). Since we have demonstrated that both the formation of C_4_ oxygenates via the aldol condensation pathway, and the reactivity of crotonaldehyde are affected by the (local) pH, we employed a constant‐current electrolysis at −10 mA cm^−2^ to identify the different reactivities of the metals. For acetaldehyde electrolysis, we found that the selectivity of Cu, Fe, Co, Ni, Ag, and Au towards 1‐butanol (Figure 4 a) and all C_4_ products (Figure S14a) can be correlated to the cathode metal‐oxygen bond strength, with Fe showing the highest selectivity towards 1‐butanol (*FE*=4.0 %). A similar trend was observed for the six above‐mentioned metals and also Pd and Pt for crotonaldehyde reduction to 1‐butanol (Figure 4 b), with Fe also showing the highest *FE* of 26.3 %. The linear‐scaling relationships end sharply in a selectivity cliff.^[23]^ The origin of the discontinuity is likely due to a phase transformation. According to their Pourbaix diagrams,^[24]^ Zn, Ti, Cr, and Mo may have surface oxide layers under the working cathodic potentials and thus have poor yields towards 1‐butanol and other C_4_ products (Section S9). Incidentally, these metals were more selective for reducing acetaldehyde to crotyl alcohol (Table S11), and crotonaldehyde to butanal (Table S12), probably because the dominating linear‐scaling relationships differ from those of the pure metals. Based on these results, we put forward that the C_4_ product selectivity is influenced by the affinity of the catalyst to oxygen.

Collectively, our results gave the reasons for the low *FE* of 1‐butanol during the electroreduction of CO_2_ on oxide‐derived copper. The first factor is the low activity for acetaldehyde production on copper materials (*FE* ${}_{\mathrm{CH}{}_{3}\mathrm{CHO}}$ ≤0.1 %, *j* ${}_{\mathrm{CH}{}_{3}\mathrm{CHO}}$ ≤ −0.41 mA cm^−2^ in Table S1, *FE* ${}_{\mathrm{CH}{}_{3}\mathrm{CHO}}$ <2.1 %, *j* ${}_{\mathrm{CH}{}_{3}\mathrm{CHO}}$ <1 mA cm^−2^ in the literature[^4^, ^25^]). The second factor is that the conversion of acetaldehyde to ethanol on copper is kinetically facile and strongly competing (*FE*_ethanol_=36.5 %, while *FE*_1‐Butanol_=9.6 % at −0.44 V vs. RHE, Figure 2 a).[^17^, ^26^] The third factor is that the formation of the C_4_ backbone and its subsequent reduction to 1‐butanol are promoted by conflicting experimental conditions. While an alkaline environment facilitates aldol condensation between acetaldehyde molecules to crotonaldehyde, its hydration to an unreactive form is also promoted.

## Conclusion

In summary, we detected for the first time C_4_ oxygenates from CO_2_ electroreduction on CuO‐derived Cu, with 1‐butanol being the most favored product amongst them (*FE*=0.056 %, *j*=*−*0.080 mA cm^−2^ at −0.48 V). The quantification was made possible by a combination of the high‐rate electrolysis, achieved by using a GDE in a flow cell, and sensitive analytical techniques. We ruled out the formation of 1‐butanol from the C−C coupling of four individual C_1_ adsorbates, such as *CO. Instead, the combination of experimental and theoretical studies established a rich reaction mechanism that combines chemical and electrochemical steps, where the electrocatalytically generated acetaldehyde plays a prominent role. Its base‐catalyzed aldol condensation, promoted by high local pH and the catalyst surface, produces crotonaldehyde, which is subsequently electroreduced to 1‐butanol.

This study further highlights the challenges associated with the one‐pot approach to converting a low molecular weight feedstock like CO_2_ into complex functionalized molecules. We discover that contrasting catalysts and conditions are required to maximize the yield of each step. In addition to operating under highly alkaline conditions, we also note that a single electrocatalytic surface can hardly optimize all the required steps. For example, among the metal discs tested, Fe was identified as the most selective catalyst for acetaldehyde reduction to 1‐butanol, but it is not active per se for CO_2_ reduction. Therefore, designing a one‐pot reactor for the electrosynthesis of large molecules would inevitably be associated with low performance. Instead, we propose that a more viable synthetic strategy would be to deconvolute the multi‐step process into sequential operation units. Therein, chemical or electrochemical reactions with different process conditions could be independently optimized. The separate stepwise‐optimized reactors could then be placed in tandem for the efficient conversion of each intermediate, leading to increased yield of the desired product.

## Conflict of interest

The authors declare no conflict of interest.

## Supporting information

As a service to our authors and readers, this journal provides supporting information supplied by the authors. Such materials are peer reviewed and may be re‐organized for online delivery, but are not copy‐edited or typeset. Technical support issues arising from supporting information (other than missing files) should be addressed to the authors.

Supplementary
