# Supplementary material for: Electrochemical Reduction of Carbon Dioxide to 1‐Butanol on Oxide‐Derived Copper
Source: Angew Chem Int Ed Engl. 2020 Sep 9;59(47):21072–9. doi: 10.1002/anie.202008289 (PMC7693243; doi:10.1002/anie.202008289)
Supplement: Supplementary file 1 — Supplementary [file ANIE-59-21072-s001.pdf]

Angewandte Chemie
Eine Zeitschrift der Gesellschaft Deutscher Chemiker

Supporting Information

Electrochemical Reduction of Carbon Dioxide to 1-Butanol on Oxide-Derived Copper

*Louisa Rui Lin Ting+, Rodrigo García-Muelas+, Antonio J. Martín+, Florentine L. P. Veenstra, Stuart Tze-Jin Chen, Yujie Peng, Edwin Yu Xuan Per, Sergio Pablo-García, Núria López, Javier Pérez-Ramírez, and Boon Siang Yeo**

anie_202008289_sm_miscellaneous_information.pdf

Table of Contents

S1 Methods ... 2

S1.1 Preparation of electrodes ... 2

S1.2 Electrolysis experiments ... 2

S1.3 Characterization of catalysts ... 3

S1.4 Nuclear magnetic resonance spectroscopic analysis ... 4

S1.5 Computational details ... 4

S2 Electrochemical CO_2 reduction on CuO-derived Cu GDE using a flow cell ... 7

S2.1 Characterization of catalysts ... 7

S2.2 CO_2 electrolysis on CuO-derived Cu ... 9

S3 Extended mechanism from CO_2 to crotonaldehyde via acetaldehyde ... 12

S4 Electrochemical acetaldehyde reduction on CuO-derived Cu ... 14

S4.1 Characterization of CuO-derived Cu deposited on Cu discs ... 14

S4.2 Acetaldehyde electroreduction in 0.1 M KOH ... 15

S4.3 Acetaldehyde electroreduction in neutral potassium phosphate buffer ... 17

S5 Electrolysis of crotonaldehyde, butanal and crotyl alcohol on CuO-derived Cu ... 19

S6 Extended mechanism from crotonaldehyde to 1-butanol ... 22

S7 Hydration of acetaldehyde and crotonaldehyde in 0.1 M KOH ... 23

S8 Acetaldehyde and crotonaldehyde electrolysis on transition metal discs ... 24

References ... 26

S1 Methods

S1.1 Preparation of electrodes

CuO gas diffusion electrode (GDE): A homogeneous Cu layer was deposited on gas diffusion layers (GDLs, Sigracet 38 BC) by magnetron sputtering using a Denton Vacuum Discovery 18 system. Radiofrequency (RF) sputtering at 100 W was performed while operating under 10 mTorr pressure and 13 cm^3 min^{-1} argon. The distance between the Cu target (> 99.99 %, Latech Scientific Supply) and the substrate was 10.16 cm. The deposited Cu layer aided in the subsequent electrodeposition process by ensuring uniform conductivity and decreased the hydrophobicity of the GDL. The electrodeposited CuO was prepared according to a previously published method.[1] The electrodeposition bath, which had a pH of 13, consisted of 30 g L^{-1} tartaric acid (> 99 %, Sigma-Aldrich), 50 g L^{-1} $CuSO_4{\cdot}5\ H_2O$ (99 %, GCE) and 70 g L^{-1} NaOH (99.0 %, GCE). A constant current of at +8 mA cm^{-2} was applied to electrodeposit CuO onto the Cu-sputtered GDLs (10 min). Prior to electrolysis, the electrodeposited CuO films were pre-reduced to CuO-derived Cu for 5 min in pure N_2-purged electrolyte at -0.6 V vs. RHE.

CuO discs: Cu discs (99.99 %, 15 mm diameter, Goodfellow) were sequentially polished with SiC paper (1200 μm, Struers), followed by 15 μm and 3 μm Diapro slurries (Struers). The discs were then sonicated in ultrapure deionized water and dried with nitrogen gas. CuO was electrodeposited onto the polished Cu discs with the above-mentioned procedure for 30 min. The pre-reduction treatment was applied as described above.

IrO_2 GDE: IrO_2 GDEs were used as anodes in the flow cells. IrO_2 nanoparticles were synthesized following a previously published procedure.[2] The synthesized colloidal suspension of IrO_2 was centrifuged at 5000 rpm for 20 min, washed twice with ultrapure water by centrifugation, dried overnight at 100 °C in air and ground into a fine powder. IrO_2 nanoparticles (30 mg) were dispersed by sonication in a solution containing 400 μL ultrapure deionized water, 400 μL isopropanol (≥ 99.5 %, Avantor) and 13.4 μL Nafion perfluorinated resin solution (5 wt.% in lower aliphatic alcohols and water, Sigma-Aldrich) to obtain a homogenized ink. This ink was hand-painted onto a GDL (9 cm^2 geometric area) and dried using a heat lamp.

S1.2 Electrolysis experiments

CO_2 electrolysis in flow cell configuration: CO_2 electrolyses were performed in a flow cell using the CuO-derived Cu GDE as the cathode (exposed geometric area: 0.72 cm^2), Ag/AgCl saturated KCl (Pine) as the reference electrode, and the IrO_2 GDE as the anode. The duration of each electrolysis was 45 min. The cathodic (2.5 mL) and anodic compartments were separated by an anion-exchange membrane (Selemion AMVN, AGC Asahi Glass). CO_2 gas (99.999 %, Linde Gas) was flowed through the back of the cathodic half-cell at a rate of 5 sccm. 1.0 M KOH (99.97 % Alfa Aesar) was pumped through the cell at 0.1 mL min^{-1} using a syringe pump. All electrochemical measurements in this paper were performed using a Gamry Reference 600 potentiostat/galvanostat and the current interrupt method was always used to compensate for the *i*R drop.

Electrolysis in H-type cell configuration: Electrolyses (1 h) of acetaldehyde (> 99.5 %, Sigma-Aldrich), crotonaldehyde (98 %, TCI), butanal (> 99.5 %, Sigma-Aldrich) and crotyl alcohol (> 97.0 %, Sigma-Aldrich) were performed in 0.1 M KOH (99.97 %, Alfa Aesar) or 0.1 M potassium phosphate buffer (pH 7), which was composed of 0.062 M K_2HPO_4 (≥ 99.0 %, Sigma-Aldrich) and 0.038 M KH_2PO_4 (≥ 99.0 %, Sigma-Aldrich). As a two-phase (liquid-electrode) interface is more suitable for the mass transport of liquid reactants to the electrode than the tri-phase (gas-liquid-electrode) interface in the flow cell, a two-compartment Teflon H-type cell separated by an anion-exchange membrane (Selemion AMVN, AGC Asahi Glass) was used for the electrolyses. The cathodic compartment (20 mL) was filled with 12 mL of electrolyte and housed the working electrode (exposed geometric surface area: 0.785 cm^2) and the reference electrode (Ag/AgCl saturated KCl, Pine). A graphite rod (Ted Pella) was used as counter electrode. The electrolyte was purged with N_2 gas at 10 sccm for 5 min prior to electrolysis and this was maintained until the end of the experiment.

Detection of products: The gaseous products (H_2, CO, CH_4, C_2H_4, C_2H_6, C_3H_6) were continuously detected from the cathodic compartment by an on-line gas chromatograph (GC, Agilent 7890A) with flame ionization detectors (FIDs) and a thermal conductivity detector (TCD). Gas products were sampled every 13.5 min. After electrolysis, alkaline catholytes were neutralized with 4 M H_2SO_4 and the liquid products were quantified with a headspace GC (HSGC, Agilent, 7890B and 7697A) and high performance liquid chromatograph (HPLC, Agilent 1260 Infinity). Aldehydes, ketones and alcohols were detected using HSGC with FID while formate and acetate were detected using HPLC with a variable wavelength detector, and 0.5 mM H_2SO_4 mobile phase. The HSGC chromatogram of the calibration standards is shown in Figure S1.

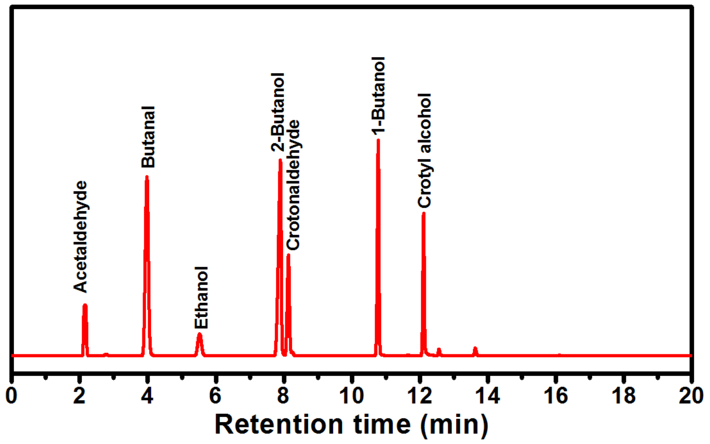

Figure S1. Headspace gas chromatogram of standard compounds in 0.1 M KOH after neutralization with 4 M H_2SO_4. The concentration of each compound is 5 mM.

S1.3 Characterization of catalysts

Scanning electron microscopy (SEM) was performed using a JEOL JSM-6701F instrument. X-ray diffraction (XRD) patterns were acquired by a Siemens 5005 instrument (Cu K_α radiation with graphite monochromator), in a locked θ-2θ scan mode from 15° to 100° 2θ with 0.1° resolution step and 1 s acquisition time per step. X-ray photoelectron spectroscopy (XPS) was measured using a Physical Electronics Quantum 2000 instrument. The applied monochromatic

Al K_α radiation (1486.6 eV) was generated from an electron beam operating at 15 kV and 32.3 W with residual pressure during measurements at 5×10^{-8} Pa. For high-resolution spectra, pass energies of 46.95 eV were used (full width at half maximum for Ag $4f_{7/2}$ = 1.0 eV at these conditions) with a neutralizer operating during analysis. The acquired spectra were analyzed without adjustment of the energy scale since no charging was observed due to their electrical conductivity.

S1.4 Nuclear magnetic resonance spectroscopic analysis

1-D ^{1}H NMR spectra with water suppression and 2-D heteronuclear single quantum coherence (HSQC) ^{1}H – ^{13}C NMR spectra were recorded on a Bruker Avance III HD 500 MHz mounted with a 5 mm BBO Prodigy (at room temperature). Phenol (7.2 ppm) and dimethylsulfoxide (2.6 ppm) were dissolved in D_2O (4.8 ppm) and added to the samples as internal standards. For the 1-D ^{1}H NMR analysis, one pulse experiment was pre-saturated on the water resonance with a π/2 pulse of 12 µs, and a recycle delay of 5 s was implemented while coadding 256 scans per experiment. These settings resulted in a high signal-to-noise ratio and high resolution per measurement. Solutions of 10 mM acetaldehyde (> 99.5 %, puriss, p.a., anhydrous, Sigma-Aldrich) and crotonaldehyde (> 99.5 %, puriss, Fluka) in ultrapure deionized water and in 0.1 M KOH were measured within a few minutes after individual preparation, unless mentioned otherwise. For the 2-D HSQC NMR analysis, an experimental pulse program for the reference compounds was set with TD 1024 and 256 fid size, with 2 scans per experiment. Solutions of the reference compounds 10 mM *n*-propanol (> 99 %, reagent grade, VWR Chemicals), 1-butanol (> 99 %, anhydrous, VWR Chemicals) and butanal (> 99.0 %, puriss, Fluka) in 0.1 M KOH were individually measured to provide a standard for signal assignment. The assignment of the unknown peak at 0.8 ppm needed 8 scans per experiment in the 2-D experiments to obtain a sufficient signal-to-noise ratio and resolution.

S1.5 Computational details

The periodic density functional theory (DFT) package VASP[3] was used to model the catalytic process, choosing the PBE[4] density functional. We included van der Waals contributions with a re-parametrized DFT-D2 method.[5-7] Inner electrons were represented by PAW pseudopotentials while the basis set for valence electrons was expanded as plane waves with a kinetic energy cut-off of 450 eV.[8] The catalyst was modeled as Cu(100), taking four metal layers, which is the most stable surface for the working potentials. The Brillouin zone was sampled by a Γ-centered k-points mesh from the Monkhorst-Pack method with a reciprocal grid size smaller than 0.03 Å^{-1}.[9] All relevant data are available from the authors. All structures are available through the ioChem-BD repository[10] (https://doi.org/10.19061/iochem-bd-1-159).

Solvation: The interaction of the solvent with the electrode is complex and would require long-time first-principles simulations to account for the fluctuations of the solvent on the surface. As these are computationally expensive approaches, simpler techniques have been put forward to study chemical processes in solution. The most successful technique, in terms of balancing cost and accuracy, requires the inclusion of few explicit water molecules that can interact with the solute or the adsorbates, while the rest are represented in a mean-field approach and

characterized by the relative permittivity of the solvent. For periodic systems, continuum solvation models have been introduced only recently.[11,12] In our case, a mixed implicit-explicit scheme was always investigated. The implicit (mean-field) solvation part was included within the VASP-MGCM model,[11,13] as it is essential to accurately describe the stability of electrochemical species.[14] In particular, anionic species in solution were computed as an ionic pair (with K^+ as the counter-cation) and an explicit water molecule, while the OH^- anion required two explicit water molecules to ensure the right basicity (Figure S2a). This method captured most of the effects from the aqueous environment. For the adsorbed species, solvation energies can be estimated only with implicit solvation within typical accuracies of 0.1 eV (Figure S2b) when compared to fully explicit water models.[13,15] In Figure S2c, we compare the results with implicit water and the mixed scheme for 2 explicit water molecules. While we observe that the accuracy was improved to 0.05 eV, it is important to note that the large number of conformations associated with these extra degrees of freedom limit our possibilities for sampling all conformations for all 30 intermediates.

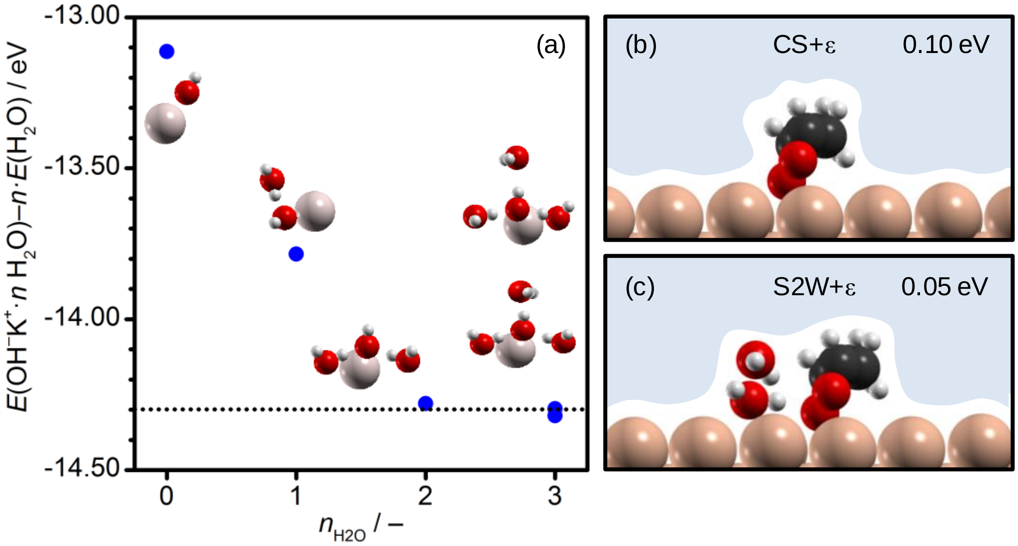

Figure S2. (a) Potential energy of a solvated $OH^-K^+\cdot n\ H_2O$ cluster, with respect to H_2O in implicit[11] solvent. For the remaining anionic species, only a water molecule and a K^+ counter-cation were needed. Solvation models derived from Ref [13]: (b) CS+ε, clean surface (CS) plus implicit solvation (ε); and (c) S2W+ε, Two explicit water molecules (S2W) plus implicit solvation (ε). In the mixed schemes represented in (b) and (c) the white region corresponds to the cavities left by the adsorbates, explicit molecules, and metal surface, while the shadowed blue region represents the volume substituted by the implicit solvent (characterized by the relative permittivity, ε). The typical error bars (0.10 and 0.05 eV) with respect to fully explicit solvent are indicated.

Computational Hydrogen Electrode: Several high-level methodologies can describe the perturbations of electric field and potential on the bonding of adsorbates.[16-18] In particular, Ref. [17] analyzed the key reactions controlling the selectivity to C_2 products, such CH_3CHO, from CO_2 reduction. However, in our case, the large number of intermediates and conformations prevents us for using these methodologies. Thus the electric potential and pH effects were introduced via the Computational Hydrogen Electrode (CHE).[19] The CHE may have large deviations for elementary steps, such as CO dimerization, which involve adsorbates with very high dipole moments.[20] Error bars in such cases are typically in the order of 0.1–0.3 eV, and require corrections related to the workfunction[16] or the number of electrons[17,21-23]. These corrections can be confidently disregarded in our study as the relevant molecules

treated are aldehydes or alcohols with small electric dipoles rather parallel to the surface. As an extreme case in the 1-butanol route, we consider atomic oxygen on Cu, (dipole of 0.52 Debye or 0.108 eÅ[24]). The potential of zero charge of Cu electrodes lies between +0.03 to −0.22 V vs. RHE at pH = 13,[25] thus our optimal working potential (–0.44 V vs. RHE) would deviate between 0.20 and 0.50 V with respect to the potential of zero charge. Assuming an the electric double layer thickness to be 3 Å,[19] the dipole contribution would be smaller than 0.02 eV (0.108 eÅ * 0.50 V / 3 Å), which is a much smaller correction than intrinsic DFT accuracy. Finally, transition state energies were estimated from linear-scaling relationships (LSR) available for Cu.[13,26] This approach can be employed as LSR hold when any combination of implicit-explicit solvation models is used.[26]

S2 Electrochemical CO_2 reduction on CuO-derived Cu GDE using a flow cell

S2.1 Characterization of catalysts

The as-deposited CuO electrodes and CuO-derived Cu electrodes after CO_2 electrolysis were characterized by SEM, XRD and XPS. SEM revealed that the as-deposited CuO films were composed of agglomerated 0.2-0.5 µm sized particles (Figure S3a). After CO_2 reduction, the catalyst retained the roughened morphology of (pre-reduced) CuO-derived Cu (Figure S3b).

As shown in Figure S3c, XRD analysis of the electrodes confirmed their chemical identities as CuO (as-deposited) and metallic Cu (after electrolysis). The latter finding is consistent with predictions from the Pourbaix diagram of the copper-water system,[27] and previous works that show the reduction of Cu oxides to metallic Cu upon the application of negative potentials necessary for CO_2 electrolysis.[28] A weak peak corresponding to Cu_2O (111) was also detected. This is likely formed from surface oxidation of the CuO-derived Cu which occurred when it was exposed to air[29] during its transfer to the XRD instrument for analysis. XPS, which probes the top few nanometers of the sample reveals that the surface of the as-deposited film is composed of CuO, as seen from the Cu $2p_{3/2}$ and $2p_{1/2}$ signals at 934.1 eV and 954.0 eV respectively, and the strong satellite peaks (Figure S3d). After electrolysis, the Cu $2p_{3/2}$ and $2p_{1/2}$ signals were shifted to 932.8 eV and 952.7 eV respectively. These values coincide with binding energy shifts of metallic Cu, and are consistent with the observation of metallic Cu in the XRD data. However, we note that Cu 2p signals of Cu_2O have similar binding energy shifts as metallic Cu, making it difficult to distinguish the two oxidation states using XPS.[29] Nonetheless, the presence of some Cu_2O on the catalyst surface would also be in agreement with Cu_2O signals in the XRD analysis. A weaker set of Cu 2p XPS signals at 934.8 eV and 954.7 eV, along with some weak satellite peaks, were also detected. These signals can be assigned to a mixture of CuO and $Cu(OH)_2$, which were also formed from the surface oxidation of CuO-derived Cu, though at a slower rate than Cu_2O.[29] The lower concentration of $Cu(OH)_2$ and CuO formed could explain why their signals were not detected in the XRD analysis. Collectively, our characterization data point to metallic Cu as the predominant component of CuO-derived Cu, after considering the influences from surface oxide formation.

Elucidating the oxidation state of copper during CO_2 reduction remains a challenge in the field. While many works have shown that copper oxides are unstable under CO_2 reduction conditions and metallic Cu is the active catalytic surface,[28,30-32] small amounts of subsurface residual oxides have also been detected[33,34]. The use of highly sensitive in-situ or even operando techniques to accurately detect and study these subsurface residual oxides in order to assess their impact on CO_2 reduction activity is still a very active area of investigation.

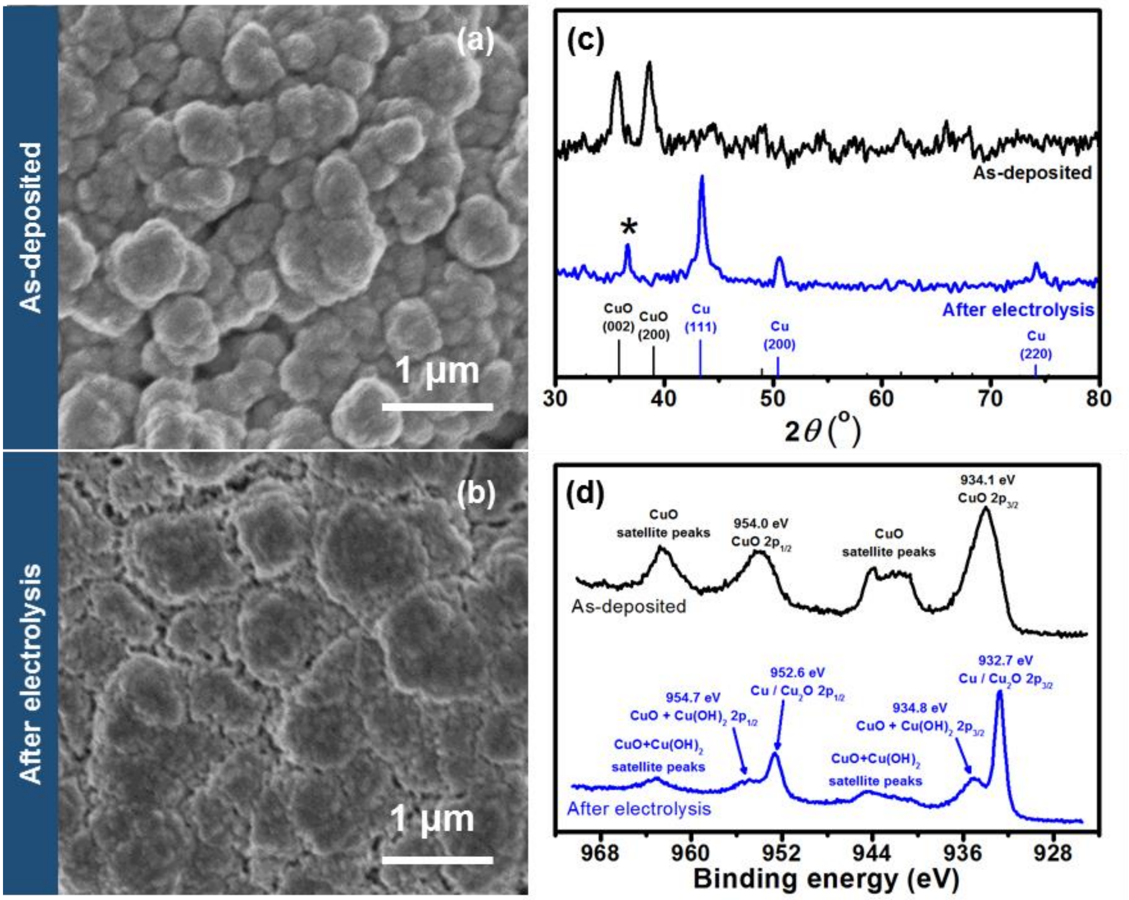

Figure S3. SEM images of (a) as-deposited CuO GDE and (b) CuO-derived Cu after CO_2 electrolysis in 1.0 M KOH at −0.48 V vs. RHE. (c) XRD patterns of as-deposited CuO GDE and CuO-derived Cu GDE after CO_2 electrolysis. Cu_2O (111) signals (indicated by *) were detected due to the surface oxidation of the catalyst. (d) Cu 2p XPS spectra of as-deposited CuO GDE and CuO-derived Cu GDE after CO_2 electrolysis.

S2.2 CO_2 electrolysis on CuO-derived Cu

We performed CO_2 electrolysis on CuO-derived Cu in a flow cell with 1.0 M KOH as the supporting electrolyte. Using a flow cell for CO_2 electrolysis enhances the mass transport of CO_2 to the catalyst, and maximizes the product yield.[35] The alkaline electrolyte promotes the production of multi-carbon molecules and suppresses the competing hydrogen evolution reaction (HER).[35] The separation of the gaseous and aqueous phases in the GDE flow cell also enables CO_2 electrolysis to be performed in an alkaline environment, as the formation of carbonates from CO_2 dissolution is suppressed.[36,37]

Table S1. Product distribution of CO_2 electroreduction in 1.0 M KOH on CuO-derived Cu GDE.

**Potential applied (V vs. RHE)**		**H_2**	**$HCOO^-$**	**CO**	**CH_4**	**CH_3OH**	**C_2H_4**	**C_2H_5OH**	**C_2H_6**	**CH_3CHO**	**C_3H_6**	***n*-PrOH**	**C_2H_5CHO**	**Allyl Alcohol**	**1-BuOH**	**Butanal**	**Total**
−0.38	*FE* (%)	47.8	10.1	27.2	N.D.	N.D.	7.9	3.9	0.10	0.10	N.D.	2.64	0.15	0.16	N.D.	N.D.	100.2
	j (mA cm^{-2})	−15.9	-3.36	−9.05	N.D.	N.D.	−2.60	−1.31	−0.03	−0.06	N.D.	−0.88	−0.05	−0.05	N.D.	N.D.	−33.3
−0.48	*FE* (%)	23.9	24.2	10.3	0.02	0.07	16.8	9.8	0.06	0.10	0.27	5.43	0.10	0.63	0.056	0.003	91.6
	j (mA cm^{-2})	−35.5	−29.3	−15.9	−0.02	−0.10	−27.4	−15.8	−0.10	−0.16	−0.44	−13.2	−0.14	−1.00	−0.080	−0.004	−147
−0.58	*FE* (%)	31.5	7.5	8.7	0.21	0.06	34.8	13.2	0.03	0.08	0.61	3.25	0.04	0.60	0.007	0.004	100.7
	j (mA cm^{-2})	−131	-24.2	−36.7	−0.83	−0.25	−152	−56.7	−0.14	−0.34	−2.55	−13.2	−0.19	−2.34	−0.026	−0.013	−418
−0.68	*FE* (%)	72.3	1.9	5.3	1.50	0.11	18.5	8.3	0.01	0.04	0.18	0.74	0.02	0.20	0.002	0.001	109.0
	j (mA cm^{-2})	−608	−14.4	−45.7	−13.5	−0.98	−162	−74.8	−0.05	−0.41	−1.56	−6.47	−0.21	−1.74	−0.013	−0.010	−850

*N.D. = not detected, *n*-PrOH = *n*-propanol, 1-BuOH= 1-butanol

We also performed control CO_2 electrolysis in 0.1 M $KHCO_3$ (99.99 %, Meryer) on CuO-derived Cu (deposited for 10 min on Cu discs) in an H-type cell (Table S2) for 45 min. C_4 oxygenates were not detected in these H-cell experiments, which had a smaller j_{CO2RR} than the flow cell experiments. This emphasizes that the high CO_2 reduction current densities generated from using the GDE in a flow cell improved the detection of minor products.

Table S2. Product distribution of CO_2 electroreduction in 0.1 M $KHCO_3$ on CuO-derived Cu discs at −0.95 V vs. RHE.

	**H_2**	**$HCOO^-$**	**CO**	**CH_4**	**CH_3OH**	**C_2H_4**	**C_2H_5OH**	**C_2H_6**	**CH_3CHO**	**CH_3COOH**	**C_3H_6**	***n*-PrOH**	**C_2H_5CHO**	**Allyl Alcohol**	**1-BuOH**	**Butanal**	**Total**
FE (%)	33.5	8.2	1.6	2.5	N.D.	28.5	9.8	0.2	0.7	0.8	N.D.	5.0	1.5	1.7	N.D.	N.D.	94.1
j (mA cm^{-2})	−9.54	−2.29	−0.43	−0.69	N.D.	−8.09	−2.83	−0.04	−0.20	−0.22	N.D.	−1.41	−0.42	−0.48	N.D.	N.D.	−28.3

*N.D. = not detected, *n*-PrOH = *n*-propanol, 1-BuOH = 1-butanol

Table S3. Comparison of CO_2 reduction activity to C_2 and C_3 products between CuO-derived Cu GDE and other Cu-loaded carbon GDEs reported in the literature.

**Electrolysis condition**	**FE_{C2} (%)**	**j_{C2} (mA cm^{-2})**	**Major C_2 product**	**FE_{C3} (%)**	**j_{C3} (mA cm^{-2})**	**Major C_3 product**	**Ref.**
−0.58 V vs. RHE in 1.0 M KOH	48.1	−201	Ethylene	4.5	−18.8	*n*-Propanol	This work
−0.79 V vs. RHE in 1.0 M KOH	65.5	−197	Ethylene	4.5	−13.5	*n*-Propanol	[35]
−0.60 V vs. RHE in 1.0 M KOH	65.3	−131	Ethylene	5.1	−10.2	*n*-Propanol	[38]
−0.80 V vs. RHE in 10 M KOH	72	−186	Ethylene	Not reported			[39]
−300 mA cm^{-2} in 1.0 M $KHCO_3$	40.1	−120	Ethylene	5.1	−15.3	*n*-Propanol	[40]
−400 mA cm^{-2} in 1.0 M KOH	45.9	−184	Ethanol	6.9	−27.6	*n*-Propanol	[41]
−275 mA cm^{-2} in 10 M KOH	83	−228	Ethylene	Not reported			[36]
−200 mA cm^{-2} in 1.0 M KOH	68.3	−137	Ethylene	Not detected			[42]

S3 Extended mechanism from CO_2 to crotonaldehyde via acetaldehyde

The mechanism to produce 1-butanol from CO_2 starts when an oxygen atom in CO_2 is protonated and subsequently removed, forming CO after two proton-coupled electron transfers. Then, a C–C bond is formed when CO couples with another CO or with HCO[17,43,44] in chemical steps strongly influenced by the electric potential and chemical environment.[20] In the first case, an oxygen atom in OCCO is protonated twice and removed as water, forming CCO, whose carbon atoms are hydrogenated to form CHCHO and then the key ethenyloxy (CH_2CHO) species,[44] which hydrogenates to form acetaldehyde (Figure S4). Alternatively, HCOCO is hydrogenated to glyoxal (CHOCHO) and glycolaldehyde ($HOCH_2CHO$) to form ethenyloxy and then acetaldehyde.[44] Finally, the C_4 backbone is formed through the aldol condensation of two acetaldehyde molecules to crotonaldehyde (Figure 2c in the main text). When reducing CO_2, the C_4 backbone may also be formed alternatively from the condensation of an adsorbed ethenyloxy species with an acetaldehyde molecule in the solution to produce 3-hydroxybutanal and crotonaldehyde (Figure S5). We outline the pathways for CO_2 reduction to ethenyloxy, acetaldehyde, and eventually crotonaldehyde in Figure S6.

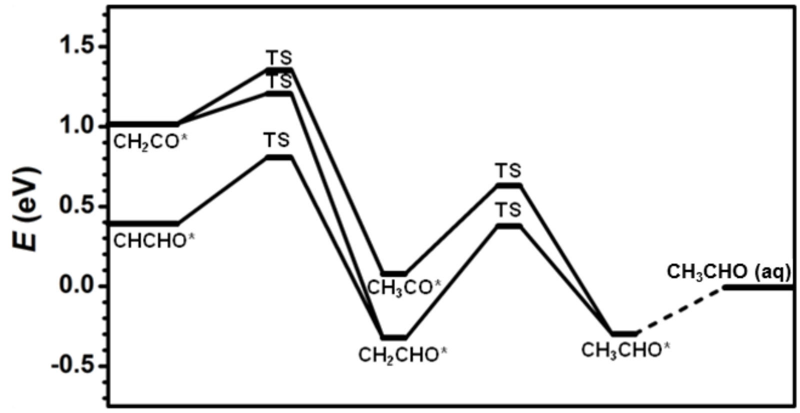

Figure S4. Potential energy diagram for acetaldehyde formation from CHCHO* and CH_2CO*. The dashed line represents the desorption of acetaldehyde. The formation of precursors CHCHO* and CH_2CO*, as well as other lateral paths, have been investigated in detail by earlier works.[1,17,43,44]

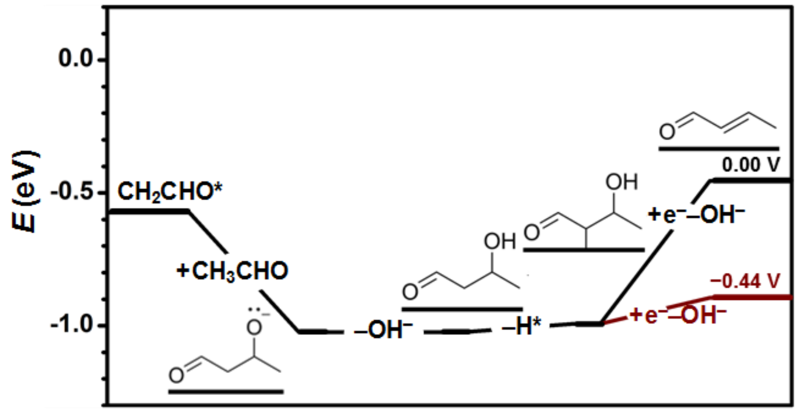

Figure S5. Potential energy diagram for the condensation of ethenyloxy with acetaldehyde to crotonaldehyde on the Cu surface (black). The final OH^- removal can be assisted by one electron donated by the surface and stabilized under reductive potentials (dark red). Water molecules were omitted for clarity. The energy references of Figure 2c are preserved, thus the energy of aqueous acetaldehyde corresponds to exactly 0.0 eV.

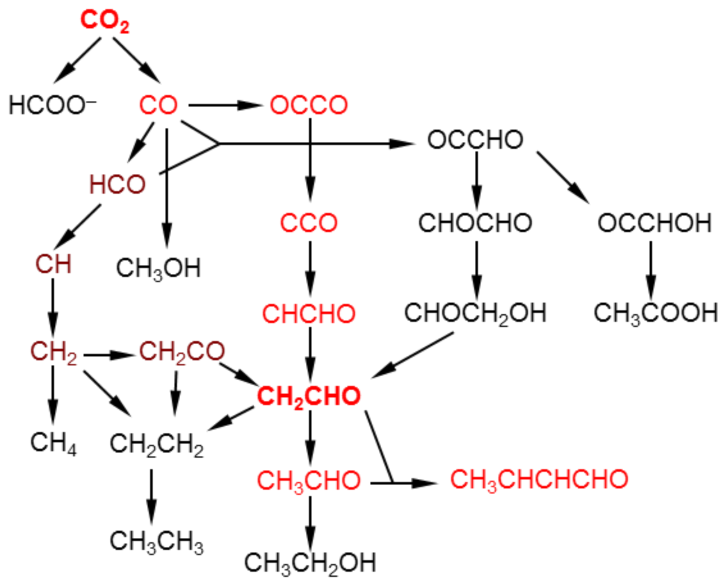

Figure S6. Main pathways from CO_2 to the key ethenyloxy intermediate (CH_2CHO, bold) and acetaldehyde, as reported in literature,[17,43,44] leading eventually to crotonaldehyde. Routes going through CHCHO[44] and ketene (CH_2CO)[1] are highlighted in red and dark red respectively. Routes involving glyoxal (CHOCHO) and glycolaldehyde ($CHOCH_2OH$) were omitted as these molecules were not detected. Paths to detected C_1 and C_2 side products are shown for completeness. Routes to crotonaldehyde from CO_2 and acetaldehyde shown in Figures S5 and Figure 2(c), respectively.

We also consider the mechanisms for CO_2 to reduction to the other detected C_1 and C_2 products in Figure S6. Among the lateral paths leading to C_1 products,[17,43,44] the carbon atom of CO_2 can be hydrogenated to produce formate. CO can also be hydrogenated to produce methanol, or stripped of its oxygen atom to produce CH_x. While CH_x species mostly evolve towards methane, CH_2 may dimerize or combine with CO to produce ethylene (CH_2CH_2) and ketene (CH_2CO) respectively.[17] CH_2CO can be hydrogenated to acetaldehyde,[1] potentially opening a third pathway towards crotonaldehyde and 1-butanol. The remaining C_2 products can also be derived from the above-mentioned intermediates. In particular, ethylene can be produced from hydrogenation and oxygen removal in both CH_2CO (derived from CO + CH_2 coupling) and CH_2CHO (derived from CO dimerization).[43,44] CH_2CH_2 and CH_3CHO can be further reduced to ethane (C_2H_6) and CH_3CH_2OH respectively, while OCCHO can be reduced to acetic acid via OCCHOH.[44]

The mechanism for CO_2 reduction to C_3 products is still under discussion, though it has been proposed that the C_3 backbone may be formed via the coupling of CO with C_2 intermediates, like COCOH or CCH_2,[45] akin to a Flory–Schulz oligomerization. The first precursor, OCCOH, can be generated from the direct protonation of the carbon monoxide dimer, OCCO. However, the latter can also be sequentially reduced and dehydrated to HOCCOH, CCOH, HCCOH, CCH, and finally the alternative C_3-precursor, CCH_2.[44]

S4 Electrochemical acetaldehyde reduction on CuO-derived Cu

S4.1 Characterization of CuO-derived Cu deposited on Cu discs

Similar to the CuO-derived Cu GDE, SEM analysis of the as-deposited samples reveals a film composed of agglomerated particles (Figure S7a). After the pre-reduction, the CuO-derived Cu film showed a roughened appearance with cracks and particles of ~0.1 µm size (Figure S7b). This morphology is retained after 1 h electrolysis of acetaldehyde in 0.1 M KOH (Figure S7c).

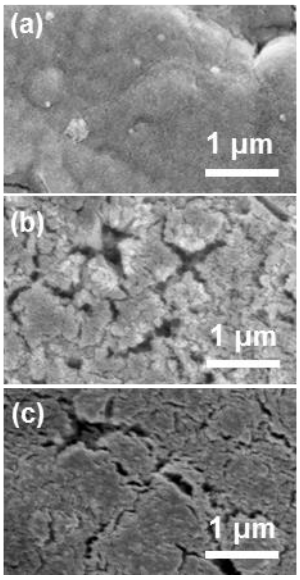

Figure S7. SEM images of the (a) as-deposited CuO, (b) CuO-derived Cu, obtained after pre-reduction of the electrodeposited CuO, and (c) CuO-derived Cu after 1 h of acetaldehyde electrolysis in 0.1 M KOH at −0.44 V vs. RHE.

S4.2 Acetaldehyde electroreduction in 0.1 M KOH

Table S4. Equations showing how various products could be formed from acetaldehyde.

Product molecule	Reaction equation	Number of electrons transferred
Ethanol, C_2H_5OH	$CH_3CHO + 2H^+ + 2e^- \rightarrow C_2H_5OH$	2
Butanal, C_3H_7CHO	$2\ CH_3CHO + 2H^+ + 2e^- \rightarrow C_3H_7CHO + H_2O$	2
Crotyl alcohol, $CH_3CH{=}CHCH_2OH$	$2\ CH_3CHO + 2H^+ + 2e^- \rightarrow CH_3CH{=}CHCH_2OH + H_2O$	2
1-Butanol, $CH_3(CH_2)_3OH$	$2\ CH_3CHO + 4H^+ + 4e^- \rightarrow CH_3(CH_2)_3OH + H_2O$	4
Crotonaldehyde, $CH_3CH{=}CHCHO$	$2\ CH_3CHO \rightarrow CH_3CH{=}CHCHO + H_2O$	0

Table S5. Product distribution of acetaldehyde electroreduction in 0.1 M KOH on CuO-derived Cu.

Applied Potential (V vs. RHE)		H_2	C_2H_6	C_2H_5OH	Crotyl alcohol	Butanal	1-Butanol	Total
−0.39	*FE* (%)	40.8	N.D.	42.3	0.22	0.37	8.45	92.1
	j (mA cm^{-2})	−2.85	N.D.	−2.81	−0.01	−0.02	−0.56	−6.79
−0.44	*FE* (%)	45.2	N.D.	36.5	0.33	0.20	9.63	91.8
	j (mA cm^{-2})	−4.98	N.D.	−4.04	−0.04	−0.02	−1.06	−11.1
−0.54	*FE* (%)	65.7	0.4	23.2	0.73	0.06	3.45	93.4
	j (mA cm^{-2})	−16.1	−0.08	−5.14	−0.17	−0.01	−0.76	−23.7
−0.64	*FE* (%)	73.3	0.7	15.5	0.67	0.03	2.00	92.2
	j (mA cm^{-2})	−26.9	−0.25	−5.70	−0.25	−0.01	−0.73	−36.7

*N.D. = not detected

After performing 1 h electrolysis of acetaldehyde in 0.1 M KOH, we observed that the anion exchange membrane had turned brown. To investigate this, we immersed one piece of membrane in solution A, which contained 0.1 M KOH and another piece in solution B, which contained 50 mM acetaldehyde in 0.1 M KOH. After immersion for 3 h, the membrane in solution A remained colorless, while the membrane in solution B turned brown (Figure S8). The liquid remained colorless in both cases. This suggests that under alkaline conditions, the acetaldehyde might have affected the membrane. We further neutralized and analyzed solution B using HSGC. The analysis reveals that, despite the membrane turning brown, there was no difference in products detected by HSGC between solution B (after membrane immersion for 3 h) and a membrane-free solution of 50 mM acetaldehyde in 0.1 M KOH aged for the same duration (Figure S9). There were also no acetaldehyde reduction products detected. This rules out the influence of the membrane on the product distribution of acetaldehyde electroreduction.

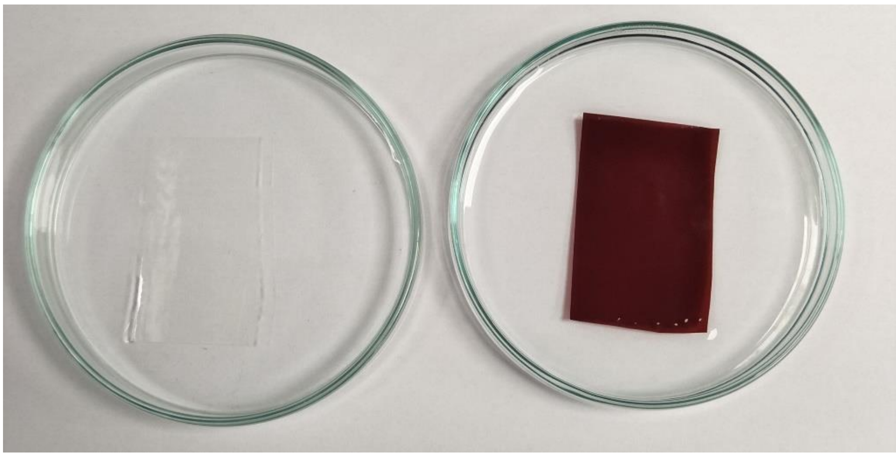

Figure S8. Photographs of Selemion AMVN anion exchange membrane in solution A (left) and solution B (right) after 3 h.

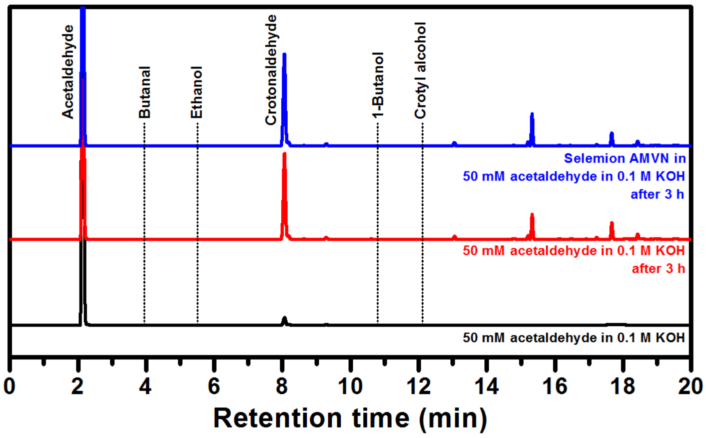

Figure S9. Headspace gas chromatogram of solution B after membrane immersion for 3 h (blue), compared with 50 mM acetaldehyde in 0.1 M KOH aged for 3 h (red) and 50 mM acetaldehyde in 0.1 M KOH, neutralized and analyzed immediately (black). The retention times of acetaldehyde reduction products are indicated with the dashed line.

S4.3 Acetaldehyde electroreduction in neutral potassium phosphate buffer

We investigated the electrolysis of acetaldehyde in 0.1 M potassium phosphate buffer (pH 7). The performance (*FE* and *j*) towards 1-butanol was significantly poorer compared to the case where 0.1 M KOH was used. The applied potentials required to generate 1-butanol were also more negative when compared to the required potentials in 0.1 M KOH.

Table S6: Product distribution of acetaldehyde electroreduction in 0.1 M potassium phosphate buffer on CuO-derived Cu.

Applied Potential (V vs. RHE)		H_2	C_2H_6	C_2H_5OH	Crotyl alcohol	Butanal	1-Butanol	Total
−0.80	*FE* (%)	26.3	0.25	68.3	N.D.	N.D.	N.D.	94.8
	j (mA cm^{-2})	−3.0	−0.03	−7.7	N.D.	N.D.	N.D.	−11.2
−0.85	*FE* (%)	32.6	0.87	61.8	N.D.	N.D.	N.D.	95.6
	j (mA cm^{-2})	−4.6	−0.12	−8.8	N.D.	N.D.	N.D.	−14.1
−1.00	*FE* (%)	53.6	1.23	41.9	N.D.	N.D.	0.01	96.8
	j (mA cm^{-2})	−15.3	−0.35	−11.9	N.D.	N.D.	−0.003	−28.5
−1.05	*FE* (%)	92.1	1.08	27.9	N.D.	N.D.	0.10	92.1
	j (mA cm^{-2})	−38.1	−0.68	−17.4	N.D.	N.D.	−0.072	−61.2

*N.D. = not detected.

During electrochemical reduction reactions, the local pH at the electrode surface is higher than that of the bulk electrolyte.[46] We therefore expect this increase in local pH to facilitate the aldol condensation and improve the production of 1-butanol. To investigate the effects of local pH on acetaldehyde reduction to 1-butanol, we performed the electrolysis under a constant applied geometric current density of −30 mA cm^{-2} in different buffer concentrations for 1 h. This was the total geometric current density observed at the onset of 1-butanol production from acetaldehyde electrolysis in 0.1 M potassium phosphate buffer.

Table S7. Product distributions of acetaldehyde electrolysis on CuO-derived Cu in 0.1 M potassium phosphate buffer at −30 mA cm^{-2}.

**Buffer concentration (M)**		**H_2**	**C_2H_6**	**C_2H_5OH**	**Crotyl alcohol**	**Butanal**	**1-Butanol**	**Total**
0.01	*FE* (%)	53.3	0.08	37.1	0.17	0.07	0.20	90.9
	j (mA cm^{-2})	−16.0	−0.02	−11.2	−0.049	−0.002	−0.061	−30
0.1	*FE* (%)	55.2	0.27	39.7	0.02	N.D.	0.01	95.3
	j (mA cm^{-2})	−16.6	−0.08	−11.9	−0.005	N.D.	−0.003	−30
1.0	*FE* (%)	82.4	0.09	13.4	N.D.	N.D.	N.D.	95.9
	j (mA cm^{-2})	−24.7	−0.03	−4.0	N.D.	N.D.	N.D.	−30

*N.D. = not detected.

S5 Electrolysis of crotonaldehyde, butanal and crotyl alcohol on CuO-derived Cu

Table S8. Half equations for relevant products obtained from crotonaldehyde reduction.

**Product molecule**	**Half-equation**	**Number of electrons transferred**
Butanal, C_3H_7CHO	$CH_3CH{=}CHCHO + 2H^+ + 2e^- \rightarrow C_3H_7CHO + H_2O$	2
Crotyl alcohol, $CH_3CH{=}CHCH_2OH$	$CH_3CH{=}CHCHO + 2H^+ + 2e^- \rightarrow CH_3CH{=}CHCH_2OH + H_2O$	2
1-Butanol, $CH_3(CH_2)_3OH$	$CH_3CH{=}CHCHO + 4H^+ + 4e^- \rightarrow CH_3(CH_2)_3OH + H_2O$	4

Table S9. Product distributions of crotonaldehyde, butanal and crotyl alcohol electrolyses on CuO-derived Cu.

**Reactant**	**Electrolyte**	**pH**	**Applied potential (V vs. RHE)**	**Applied potential (V vs. SHE)**		**H_2**	**C_2H_5OH**	**Crotyl alcohol**	**Butanal**	**1-Butanol**	**Total**
Crotonaldehyde, $CH_3CH{=}CHCHO$	0.1 M KOH	13.0	-0.44	-1.20	*FE* (%)	52.9	4.5	1.1	0.3	14.8	72.2
					j (mA cm^{-2})	−7.08	−0.76	−0.12	−0.05	−2.55	−18.5
	0.1 M potassium phosphate buffer	7.0	-0.44	-0.90	*FE* (%)	0.5	N.D.	3.2	77.9	3.9	85.6
					j (mA cm^{-2})	-0.03	N.D.	-0.18	-4.29	-0.22	-5.51
	0.1 M potassium phosphate buffer	7.0	-0.79	-1.20	*FE* (%)	31.1	N.D.	5.4	7.0	46.9	90.3
					j (mA cm^{-2})	−8.04	N.D.	−1.33	−1.72	−11.7	−25.4
Butanal, C_3H_7CHO	0.1 M KOH	13.0	-0.44	-1.20	*FE* (%)	70.3	N.D.	N.D.	N.A.	17.3	87.6
					j (mA cm^{-2})	−5.36	N.D.	N.D.	N.A.	−1.61	−7.58
	0.1 M potassium phosphate buffer	7.0	-0.79	-1.20	*FE* (%)	45.7	N.D.	N.D.	N.A.	45.8	91.6
					j (mA cm^{-2})	−6.89	N.D.	N.D.	N.A.	−6.87	−14.9
Crotyl alcohol, $CH_3CH{=}CHCH_2OH$	0.1 M KOH	13.0	-0.44	-1.20	*FE* (%)	88.4	N.D.	N.A.	N.D.	N.D.	88.4
					j (mA cm^{-2})	−3.96	N.D.	N.A.	N.D.	N.D.	−4.50

*N.D. = not detected, N.A. = not applicable.

Table S10. Total C_4 Faradaic efficiency and relative Faradaic selectivity toward C_4 products from electrolysis of acetaldehyde and crotonaldehyde on CuO-derived Cu in 0.1 M KOH at −0.44 V vs. RHE.

Reactant	$FE_{all\ C4}$ (%)	Relative Faradaic selectivity (%)		
		Crotyl alcohol	Butanal	1-Butanol
Acetaldehyde	10.2	3.3	2.0	94.7
Crotonaldehyde	16.2	6.6	1.9	91.4

Relative Faradaic selectivity $= FE_{particular\ C4\ product} / FE_{all\ C4}$

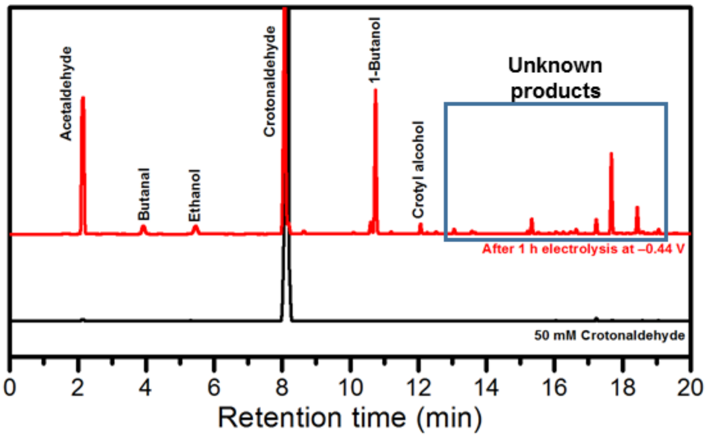

Figure S10. Headspace gas chromatogram of 50 mM crotonaldehyde in 0.1 M KOH (black) and the electrolyte mixture after 1 h electrolysis at −0.44 V vs. RHE (red). Both samples were neutralized with 4 M H_2SO_4 prior to analysis.

S6 Extended mechanism from crotonaldehyde to 1-butanol

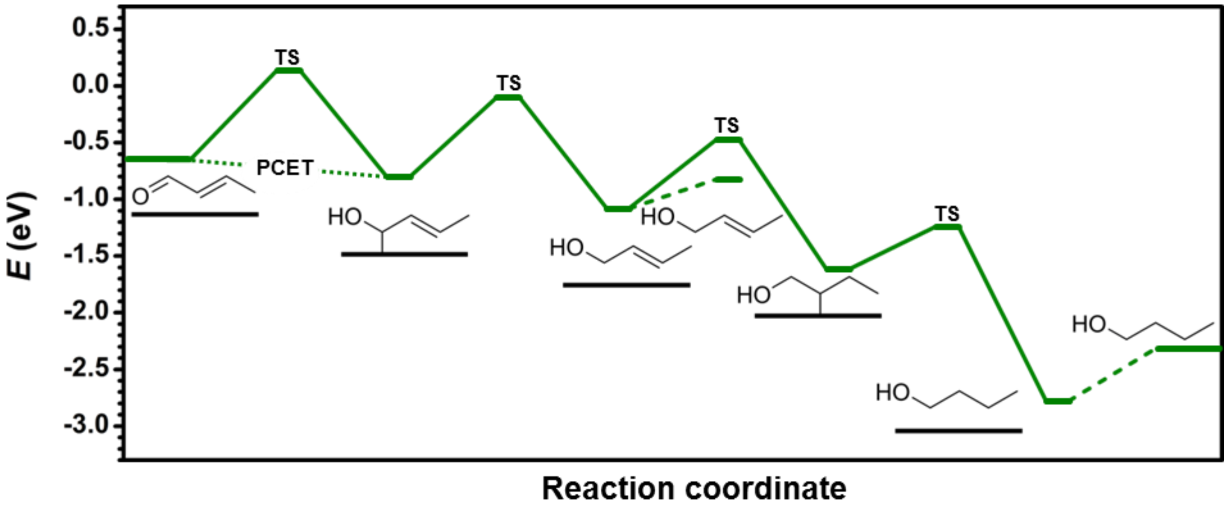

Figure S11. Potential energy diagram for the reduction of crotonaldehyde to 1-butanol via crotyl alcohol. The dashed lines represent desorption processes. The dotted lines represent proton-coupled electron transfers (PCET). Under negative potentials, the O atom of crotonaldehyde can be hydrogenated via PCET. The remaining reactions are chemical steps. Once formed, crotyl alcohol would desorb rather than react.

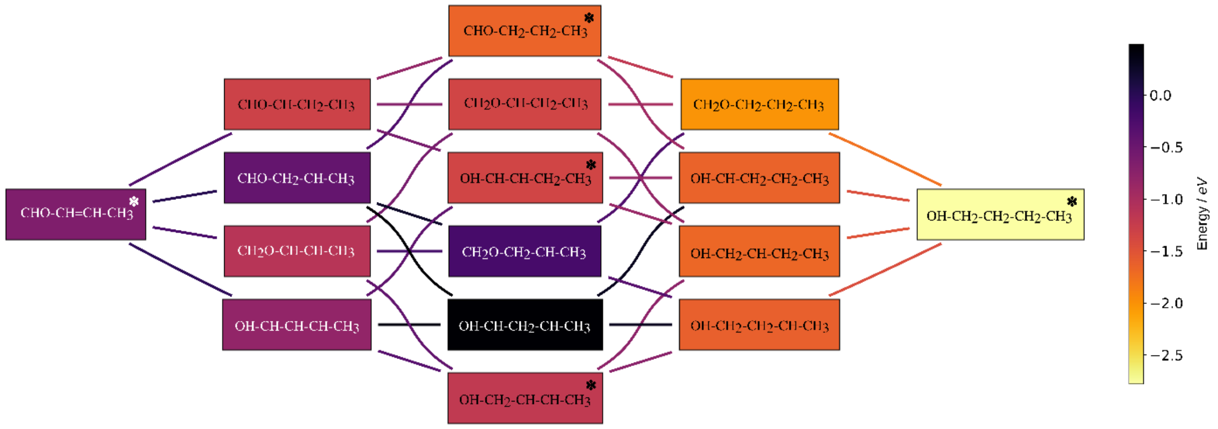

Figure S12. Energy profile of crotonaldehyde reduction to 1-butanol via chemical steps. The color of each box, with reference to the scale on the right, represents the energy of each intermediate. Lines represent transition state energies between the connected intermediates. Butanal ($CHO\text{-}CH_2\text{-}CH_2\text{-}CH_3$) is the most favored intermediate both thermodynamically and kinetically. Species that are stable in aqueous phase are marked with a ✻ symbol at the top-right of the box. Adsorbed hydrogen species (H*) have been omitted for simplicity.

S7 Hydration of acetaldehyde and crotonaldehyde in 0.1 M KOH

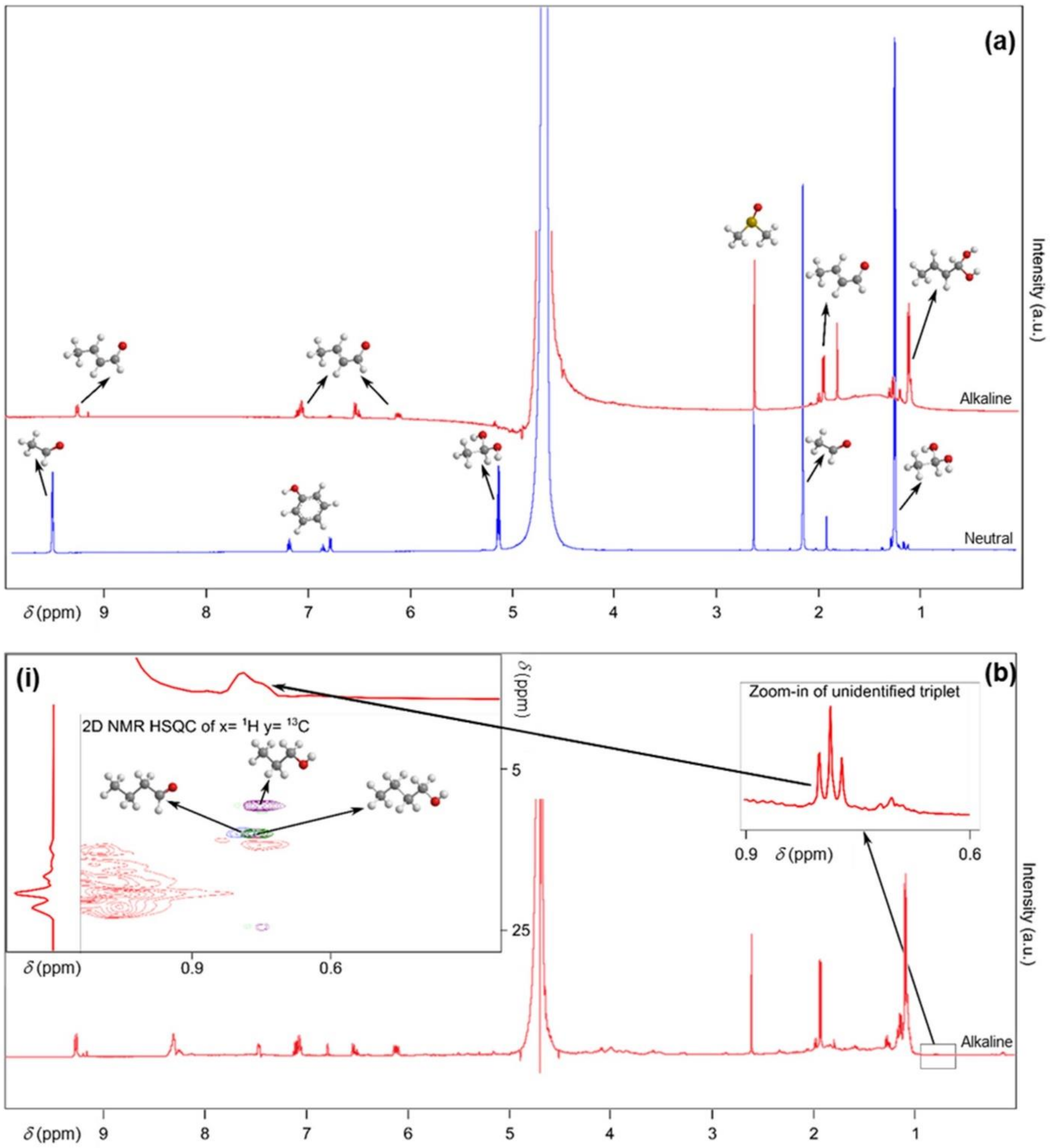

Figure S13. Nuclear magnetic resonance (NMR) spectroscopic analyses (δ refers to the chemical shift). (a) ^{1}H NMR spectra of fresh (< 2 min) solutions of 10 mM acetaldehyde in ultrapure deionized water (blue) and 0.1 M KOH (red). The conversion of acetaldehyde to hydrated acetaldehyde in water is evidenced by the ~1:1 ratio of their respective peaks. In alkaline conditions, crotonaldehyde and hydrated crotonaldehyde are the predominant compounds, consistent with the aldol condensation of acetaldehyde to crotonaldehyde, followed by its hydration. (b) NMR spectra of 10 mM crotonaldehyde in 0.1 M KOH after 50 min. The chemical reactions of crotonaldehyde in the alkaline environment led to multiple unidentified peaks, including the CH_3 triplet peak at 0.8 ppm which suggests the formation of C_4 alcohols or aldehydes. (Inset i) Two-dimensional heteronuclear single quantum coherence (HSQC) spectra of 10 mM *n*-propanol, butanal and 1-butanol in 0.1 M KOH overlaid with the spectrum of 10 mM crotonaldehyde in 0.1 M KOH. The non-overlap of the signals at ~0.8 ppm indicates that neither *n*-propanol, butanal nor 1-butanol were formed through a chemical route.

S8 Acetaldehyde and crotonaldehyde electrolysis on transition metal discs

The ability of a series of transition metal discs to catalyze acetaldehyde reduction to 1-butanol was studied. The discs were polished sequentially with SiC paper (1200 µm, Struers) followed by 15 µm and 3 µm Diapro slurries (Struers). They were then sonicated in ultrapure water and dried with nitrogen gas. Galvanostatic electrolyses (1 h) were performed at −10 mA cm^{-2} in 50 mM acetaldehyde in 0.1 M KOH. The constant-current electrolysis helps to minimize the differences in local pH between the different metals used. Nonetheless, we note that some electrolysis products like ethanol are weak acids, and the difference in their production rates among the different metals may potentially result in small differences on local pH between metals, impacting the actual amounts of crotonaldehyde present (and further reduced) at the electrode surface. Thus, we also include the Faradaic efficiency ratio of 1-butanol to C_4 products (i.e. $FE_{1\text{-butanol}}$ / FE_{C4}) as a fairer basis of comparison between the different metals.

Table S11. Product distribution of 50 mM acetaldehyde electrolysis in 0.1 M KOH on metal discs at −10 mA cm^{-2}.

Metal	Faradaic efficiency (%)							
	H_2	C_2H_6	C_2H_5OH	Crotyl alcohol	Butanal	1-Butanol	Total	1-Butanol / C_4
Cu	70.1	0.4	18.6	1.4	0.2	1.7	92.3	0.52
Fe	71.0	0.7	19.0	0.4	0.2	4.0	95.2	0.88
Co	73.0	0.5	15.6	0.4	0.4	2.7	92.7	0.76
Ni	76.3	0.2	11.5	0.6	0.4	1.4	90.2	0.59
Ag	75.1	1.2	10.5	1.3	0.1	0.5	88.8	0.26
Au	75.0	0.5	10.4	0.1	0.02	0.1	86.1	0.41
Pt	89.3	0.3	1.9	0.2	0.1	N.D.	91.8	N.A.
Pd	4.7	N.D.	2.1	0.5	0.4	N.D.	7.7	N.A.
Zn	63.9	0.2	26.9	0.2	0.01	0.2	91.4	0.50
Ti	93.7	0.1	2.1	0.1	0.08	N.D.	96.2	N.A.
Cr	98.6	0.02	0.7	0.04	0.02	N.D.	99.4	N.A.
Mo	95.1	N.D.	1.0	0.2	0.1	N.D.	96.3	N.A.

*N.D. = not detected. We note that the low total *FE* on Pd is due to its hydrogen absorption behavior.

We also performed one-hour crotonaldehyde electrolysis on the metal discs in 0.1 M potassium phosphate buffer at −10 mA cm^{-2}. The neutral buffer was used as a supporting electrolyte to retard the hydration of crotonaldehyde to an unreactive diol.

Table S12. Product distribution of 50 mM crotonaldehyde electrolysis in 0.1 M potassium phosphate buffer on metal discs at −10 mA cm^{-2}.

Metal	Faradaic efficiency (%)					
	H_2	Crotyl alcohol	Butanal	1-Butanol	Total	1-Butanol / C_4
Cu	40.7	5.6	22.5	12.4	81.1	0.31
Fe	35.9	5.3	13.0	26.3	80.4	0.59
Co	45.7	5.1	19.9	6.8	77.5	0.21
Ni	41.3	5.4	38.9	6.0	91.6	0.12
Ag	29.6	25.2	16.3	4.1	75.1	0.09
Au	39.1	5.9	16.5	3.9	65.4	0.24
Pt	81.0	1.6	7.9	1.6	92.1	0.15
Pd	2.2	2.7	21.9	1.4	28.2	0.05
Zn	38.9	1.3	2.5	0.8	43.5	0.18
Ti	54.6	1.9	7.0	0.7	64.3	0.07
Cr	72.1	0.8	5.5	0.5	79.9	0.06
Mo	87.0	0.8	2.2	0.1	90.1	0.04

*N.D. = not detected; we note that the low total *FE* could be due to undetected products.

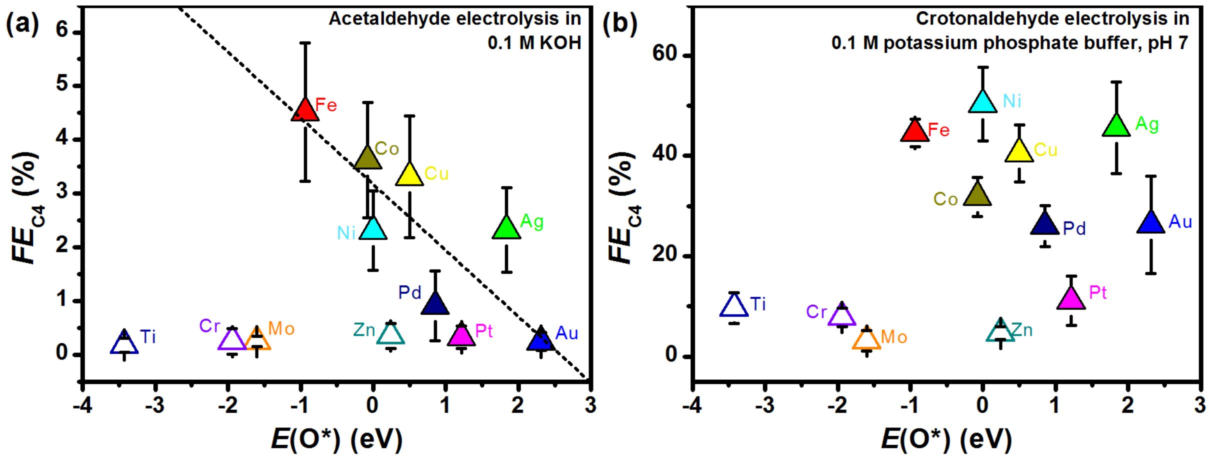

Figure S14. Faradaic efficiency of C_4 oxygenates from −10 mA cm^{-2} constant-current electrolysis of (a) acetaldehyde and (b) crotonaldehyde on selected metals as a function of the DFT-computed adsorbed oxygen stability on these metals with respect to water and hydrogen. Metals that are typically oxides at 0 V vs. RHE at the pH of the supporting electrolyte are shown as hollow symbols. The oxide layer present on the surface of these metals might have prevented the adsorption of acetaldehyde and crotonaldehyde, which resulted in poor C_4 selectivity.

References

[1] D. Ren, J. Fong, B. S. Yeo, *Nat. Commun.* **2018**, *9*, 925.
[2] K. Chakrapani, S. Sampath, *Chem. Commun.* **2015**, *51*, 9690-9693.
[3] G. Kresse, J. Furthmüller, *Comp. Mater. Sci.* **1996**, *6*, 15-50.
[4] J. P. Perdew, K. Burke, M. Ernzerhof, *Phys. Rev. Lett.* **1996**, *77*, 3865-3868.
[5] S. Grimme, *J. Comput. Chem.* **2006**, *27*, 1787-1799.
[6] T. Bučko, J. Hafner, S. Lebègue, J. G. Ángyán, *J. Phys. Chem. A* **2010**, *114*, 11814-11824.
[7] N. Almora-Barrios, G. Carchini, P. Błoński, N. López, *J. Chem. Theory Comput.* **2014**, *10*, 5002-5009.
[8] P. E. Blöchl, *Phys. Rev. B* **1994**, *50*, 17953-17979.
[9] H. J. Monkhorst, J. D. Pack, *Phys. Rev. B* **1976**, *13*, 5188-5192.
[10] M. Álvarez-Moreno, C. de Graaf, N. López, F. Maseras, J. M. Poblet, C. Bo, *J. Chem. Inf. Model.* **2015**, *55*, 95-103.
[11] M. Garcia-Ratés, N. López, *J. Chem. Theory Comput.* **2016**, *12*, 1331-1341.
[12] K. Mathew, R. Sundararaman, K. Letchworth-Weaver, T. A. Arias, R. G. Hennig, *J. Chem. Phys.* **2014**, *140*, 084106.
[13] M. Garcia-Ratés, R. García-Muelas, N. López, *J. Phys. Chem. C* **2017**, *121*, 13803-13809.
[14] R. García-Muelas, F. Dattila, T. Shinagawa, A. J. Martín, J. Pérez-Ramírez, N. López, *J. Phys. Chem. Lett.* **2018**, *9*, 7153-7159.
[15] L. Bellarosa, R. García-Muelas, G. Revilla-López, N. López, *ACS Cent. Sci.* **2016**, *2*, 109-116.
[16] K. Chan, J. K. Nørskov, *J. Phys. Chem. Lett.* **2016**, *7*, 1686-1690.
[17] A. J. Garza, A. T. Bell, M. Head-Gordon, *ACS Catal.* **2018**, *8*, 1490-1499.
[18] T. Cheng, H. Xiao, W. A. Goddard, *Proc Natl Acad Sci* **2017**, *114*, 1795.
[19] J. K. Nørskov, J. Rossmeisl, A. Logadottir, L. Lindqvist, J. R. Kitchin, T. Bligaard, H. Jónsson, *J. Phys. Chem. B* **2004**, *108*, 17886-17892.
[20] R. B. Sandberg, J. H. Montoya, K. Chan, J. K. Nørskov, *Surf. Sci.* **2016**, *654*, 56-62.
[21] G. Kastlunger, P. Lindgren, A. A. Peterson, *J. Phys. Chem. C* **2018**, *122*, 12771-12781.
[22] L. D. Chen, M. Bajdich, J. M. P. Martirez, C. M. Krauter, J. A. Gauthier, E. A. Carter, A. C. Luntz, K. Chan, J. K. Nørskov, *Nat. Commun.* **2018**, *9*, 3202.
[23] P. Lindgren, G. Kastlunger, A. A. Peterson, *ACS Catal.* **2020**, *10*, 121-128.
[24] X. Duan, O. Warschkow, A. Soon, B. Delley, C. Stampfl, *Phys. Rev. B* **2010**, *81*, 075430.
[25] A. Łukomska, J. Sobkowski, *J. Electroanal. Chem.* **2004**, *567*, 95-102.
[26] Q. Li, R. García-Muelas, N. López, *Nat. Commun.* **2018**, *9*, 526.
[27] B. Beverskog, I. Puigdomenech, *J. Electrochem. Soc.* **1997**, *144*, 3476-3483.
[28] D. Ren, Y. Deng, A. D. Handoko, C. S. Chen, S. Malkhandi, B. S. Yeo, *ACS Catal.* **2015**, *5*, 2814-2821.
[29] I. Platzman, R. Brener, H. Haick, R. Tannenbaum, *J. Phys. Chem. C* **2008**, *112*, 1101-1108.
[30] L. Mandal, K. R. Yang, M. R. Motapothula, D. Ren, P. Lobaccaro, A. Patra, M. Sherburne, V. S. Batista, B. S. Yeo, J. W. Ager, J. Martin, T. Venkatesan, *ACS Appl. Mater. Interfaces* **2018**, *10*, 8574-8584.
[31] A. J. Garza, A. T. Bell, M. Head-Gordon, *J. Phys. Chem. Lett.* **2018**, *9*, 601-606.
[32] F. Scholten, I. Sinev, M. Bernal, B. Roldan Cuenya, *ACS Catal.* **2019**, *9*, 5496-5502.
[33] A. Eilert, F. Cavalca, F. S. Roberts, J. Osterwalder, C. Liu, M. Favaro, E. J. Crumlin, H. Ogasawara, D. Friebel, L. G. M. Pettersson, A. Nilsson, *J. Phys. Chem. Lett.* **2017**, *8*, 285-290.
[34] F. Cavalca, R. Ferragut, S. Aghion, A. Eilert, O. Diaz-Morales, C. Liu, A. L. Koh, T. W. Hansen, L. G. M. Pettersson, A. Nilsson, *J. Phys. Chem. C* **2017**, *121*, 25003-25009.
[35] S. Ma, M. Sadakiyo, R. Luo, M. Heima, M. Yamauchi, P. J. A. Kenis, *J. Power Sources* **2016**, *301*, 219-228.

[36] C.-T. Dinh, T. Burdyny, M. G. Kibria, A. Seifitokaldani, C. M. Gabardo, F. P. G. de Arquer, A. Kiani, J. P. Edwards, P. De Luna, O. S. Bushuyev, *Science* **2018**, *360*, 783-787.
[37] S. Malkhandi, B. S. Yeo, *Curr. Opin. Chem. Eng.* **2019**, *26*, 112-121.
[38] T. T. H. Hoang, S. Ma, J. I. Gold, P. J. A. Kenis, A. A. Gewirth, *ACS Catal.* **2017**, *7*, 3313-3321.
[39] Y. Wang, H. Shen, K. J. T. Livi, D. Raciti, H. Zong, J. Gregg, M. Onadeko, Y. Wan, A. Watson, C. Wang, *Nano Lett.* **2019**, *19*, 8461-8468.
[40] N. S. Romero Cuellar, K. Wiesner-Fleischer, M. Fleischer, A. Rucki, O. Hinrichsen, *Electrochim. Acta* **2019**, *307*, 164-175.
[41] T.-T. Zhuang, Z.-Q. Liang, A. Seifitokaldani, Y. Li, P. De Luna, T. Burdyny, F. Che, F. Meng, Y. Min, R. Quintero-Bermudez, C. T. Dinh, Y. Pang, M. Zhong, B. Zhang, J. Li, P.-N. Chen, X.-L. Zheng, H. Liang, W.-N. Ge, B.-J. Ye, D. Sinton, S.-H. Yu, E. H. Sargent, *Nat. Catal.* **2018**, *1*, 421-428.
[42] G. L. De Gregorio, T. Burdyny, A. Loiudice, P. Iyengar, W. A. Smith, R. Buonsanti, *ACS Catal.* **2020**, *10*, 4854-4862.
[43] R. Kortlever, J. Shen, K. J. P. Schouten, F. Calle-Vallejo, M. T. M. Koper, *J. Phys. Chem. Lett.* **2015**, *6*, 4073-4082.
[44] S. Nitopi, E. Bertheussen, S. B. Scott, X. Liu, A. K. Engstfeld, S. Horch, B. Seger, I. E. L. Stephens, K. Chan, C. Hahn, J. K. Nørskov, T. F. Jaramillo, I. Chorkendorff, *Chem. Rev.* **2019**, *119*, 7610-7672.
[45] J. Li, F. Che, Y. Pang, C. Zou, J. Y. Howe, T. Burdyny, J. P. Edwards, Y. Wang, F. Li, Z. Wang, P. De Luna, C.-T. Dinh, T.-T. Zhuang, M. I. Saidaminov, S. Cheng, T. Wu, Y. Z. Finfrock, L. Ma, S.-H. Hsieh, Y.-S. Liu, G. A. Botton, W.-F. Pong, X. Du, J. Guo, T.-K. Sham, E. H. Sargent, D. Sinton, *Nat. Commun.* **2018**, *9*, 4614.
[46] H. Ooka, M. C. Figueiredo, M. T. M. Koper, *Langmuir* **2017**, *33*, 9307-9313.
